# Evolution of genetic networks for human creativity

**DOI:** 10.1038/s41380-021-01097-y

**Published:** 2021-04-21

**Authors:** I. Zwir, C. Del-Val, M. Hintsanen, K. M. Cloninger, R. Romero-Zaliz, A. Mesa, J. Arnedo, R. Salas, G. F. Poblete, E. Raitoharju, O. Raitakari, L. Keltikangas-Järvinen, G. A. de Erausquin, I. Tattersall, T. Lehtimäki, C. R. Cloninger

**Affiliations:** 1 Department of Psychiatry, Washington University School of Medicine, St. Louis, MO USA; 2 Department of Computer Science and Artificial Intelligence, University of Granada, Andalusian Research Institute in Data Science and Computational Intelligence, Granada, Spain; 3 Unit of Psychology, Faculty of Education, University of Oulu, Oulu, Finland; 4 Anthropedia Foundation, St. Louis, MO USA; 5 The Menninger Clinic, Baylor College of Medicine, and DeBakey VA Medical Center, Houston, TX USA; 6 The Menninger Clinic, Houston, TX USA; 7 Department of Clinical Chemistry, Fimlab Laboratories, and Finnish Cardiovascular Research Center - Tampere, Faculty of Medicine and Health Technology, Tampere University, Tampere, Finland; 8 Center for Population Health Research, University of Turku and Turku University Hospital; Research Center of Applied and Preventive Cardiovascular Medicine, University of Turku; Department of Clinical Physiology and Nuclear Medicine, Turku University Hospital, Turku, Finland; 9 Department of Psychology and Logopedics, University of Helsinki, Helsinki, Finland; 10 Department of Psychiatry, University of Texas San Antonio, Long School of Medicine, The Glenn Briggs Institute of Alzheimer’s and Neurodegenerative Disorders, San Antonio, TX USA; 11 American Museum of Natural History, New York, NY USA

**Keywords:** Genetics, Psychology, Neuroscience

## Abstract

The genetic basis for the emergence of creativity in modern humans remains a mystery despite sequencing the genomes of chimpanzees and Neanderthals, our closest hominid relatives. Data-driven methods allowed us to uncover networks of genes distinguishing the three major systems of modern human personality and adaptability: emotional reactivity, self-control, and self-awareness. Now we have identified which of these genes are present in chimpanzees and Neanderthals. We replicated our findings in separate analyses of three high-coverage genomes of Neanderthals. We found that Neanderthals had nearly the same genes for emotional reactivity as chimpanzees, and they were intermediate between modern humans and chimpanzees in their numbers of genes for both self-control and self-awareness. 95% of the 267 genes we found only in modern humans were not protein-coding, including many long-non-coding RNAs in the self-awareness network. These genes may have arisen by positive selection for the characteristics of human well-being and behavioral modernity, including creativity, prosocial behavior, and healthy longevity. The genes that cluster in association with those found only in modern humans are over-expressed in brain regions involved in human self-awareness and creativity, including late-myelinating and phylogenetically recent regions of neocortex for autobiographical memory in frontal, parietal, and temporal regions, as well as related components of cortico-thalamo-ponto-cerebellar-cortical and cortico-striato-cortical loops. We conclude that modern humans have more than 200 unique non-protein-coding genes regulating co-expression of many more protein-coding genes in coordinated networks that underlie their capacities for self-awareness, creativity, prosocial behavior, and healthy longevity, which are not found in chimpanzees or Neanderthals.

## Introduction

One of the most fundamental questions about human nature is what sparked the explosive emergence of creativity in modern humans before their widespread dispersal from Africa and the subsequent extinction of Neanderthals [1–4]. Major controversies persist about the basis for human creativity in art and science, as well as about the differences in cognition, language, and personality that distinguish modern humans from extinct hominids [5–8]. These controversies occur because the archeological and fossil records are incomplete and subject to ambiguous interpretation [9, 10].

## What distinguishes behaviorally modern humans from other hominids?

Anthropologists distinguish behaviorally modern *Homo sapiens* (Sapiens) from other hominids by virtue of Sapiens’ enhanced cognitive, social, and physical adaptability. Behaviorally modern Sapiens demonstrate remarkable creativity compared to other hominids: that is, they show signs of innovation, flexibility, depth of planning, and related cognitive abilities for symbolism and self-awareness that enable spontaneous generation of narrative art and language [2, 5, 11–13]. Early behaviorally modern Sapiens were also more prosocial in their behaviors than archaic hominids: they maintained larger social groups, established reciprocal social networks for remote trade, and regularly cooperated with one another in groups composed partially or completely of non-kin, as well as providing altruistic support and cooperation with non-kin who were raising children or disabled [11, 14, 15]. Behaviorally modern Sapiens are also distinguished by their healthy longevity, as evidenced by their resilience to cold and other climatic extremes [16], lower energy requirements and reduced mortality from injury and disease [17–19], and a prolonged post-reproductive lifespan that facilitates cooperative breeding [11, 20, 21], which have all enhanced health and viability in diverse, harsh, and unpredictable habitats throughout the world.

The lineages of Sapiens and *Homo neanderthalensis* (Neanderthals) are thought to have diverged from a common ancestor during the Middle Pleistocene before 500 thousand years ago (kya), at a time when the lineage of Sapiens was isolated in Africa and that of Neanderthals was confined to Europe and western Asia [22, 23]. Precursor forms to Neanderthals are recognized at least by 430 kya in Europe [24], but the behaviors and genomes of Neanderthals themselves are best known from artifacts and fossils dating from 130 to 40 kya in Eurasia [Supplementary Information]. In contrast, anatomically modern Sapiens emerged in eastern Africa [22] by 200 to 160 kya [2, 25] following a period from 320 to 200 kya marked by unpredictable climactic fluctuations [15, 25, 26] that were superimposed on a long-term pattern of progressive aridity [27]. Under these challenging ecological conditions, precursors of behaviorally modern Sapiens began to maintain larger social groups and reciprocal social alliances with non-kin (e.g., remote trade networks), express themselves symbolically (e.g., art, ornamentation), collect remote resources (e.g., pigments, obsidian, and other special stones) for later use, flexibly use expanded dietary options (e.g., fishing and collecting shellfish) in times of unpredictable resource availability, and began to accumulate cultural knowledge and standardized technologies that enhanced their adaptability and well-being [15, 25, 26, 28]. Recent findings, however, suggest that behaviorally modern Sapiens, with a distinctively more imaginative and flexible set of abilities that had not been observed in any hominids there or elsewhere, emerged in Africa about 100 kya and spread throughout the continent thereafter [29].

The ecological and economic pressure on the smaller bands of mobile and muscular hunters in the lineage of the Neanderthals in Europe were different from those on the lineage of Sapiens in East Africa [30]. Neanderthals and their European ancestors were less resilient to climatic extremes, particularly cold, and their hunting of large land animals demanded high daily energy expenditures [31]. Nevertheless, Neanderthals were able to function successfully before they had to compete with Sapiens. Neanderthals were able to conserve their reliance on hunting large land animals by moving out of inhospitable areas without the need to develop greater social connectedness or more efficient and diverse technologies and resources [17, 22, 28, 31].

The innovative practices of Sapiens are best documented after 50 kya when they flourished to a stage in which the creative imagination of fully modern humans was unmistakably displayed [2, 5, 32]. The flourishing of behavioral modernity in the late Pleistocene is likely to have been facilitated by incremental cultural and neurobiological processes by which complex behaviors like narrative figural art and language emerged by exaptation and behavioral recruitment [2]. In any case, the basic features of behavioral modernity must have been already present when Sapiens spread out of Africa between 65 and 55 kya, while the African climate became drier and colder [27, 33]. The inventive, sociable, and resilient Sapiens were able to adapt well to unpredictable and diverse conditions as they migrated out of Africa and spread throughout the world, replacing all other hominids by 40 kya and producing cultures that flourished by continuing to expand in knowledge, art, science, technology, and population density to the present day.

## What is creativity? How is it measured?

The most distinctive and prominent feature of behavioral modernity identified by paleoanthropologists and archeologists is what psychologists have described as creativity, particularly the achievements and personality traits of highly creative people. Creativity can be succinctly defined as the use of imagination or original ideas to achieve valued goals [34, 35], and is a multifaceted phenomenon that can be assessed in terms of particular aspects of intelligence and/or particular aspects of personality [36–38]. The psychometric tests of the creative aspects of intelligence were developed by Guilford and Torrance to measure aspects of divergent thinking in verbal and pictorial tasks. Divergent thinking is an innovative way of solving problems by exploring many possible solutions, making spontaneous intuitive connections among what are conventionally regarded as disparate phenomena, while tolerating some ambiguity [39]. Divergent thinking typically occurs in states of restful and playful self-aware evaluation of internal thoughts and images, such as mind-wandering in the default mode, flow, free association, day-dreaming, or contemplation [37, 40–42], which depends on activation of the medial prefrontal cortex for evaluation of internal stimuli as a core component of the self-awareness network [43, 44]. In contrast, convergent thinking follows a logical sequence of inferences to arrive at a single solution with certainty; it depends on the lateral prefrontal and parietal cortices, which are core components of the executive self-control network that supports purposeful use of symbols and intentional inhibition of externally triggered impulses [19, 45, 46].

The features used to measure divergent thinking include originality (inventive and imaginative thoughts), flexibility (ability to move from one conceptual field to another), fluency (free-flow of many relevant ideas and responses), elaboration (many vivid, specific details), a high degree of abstraction, and persistence despite uncertainty [39, 47, 48]. Divergent thinking tests developed by Guilford and Torrance are the most widely used tests of creative intellectual functioning because they are strongly predictive of creative achievement and problem-solving ability in everyday life [38, 47, 48].

## How is creativity related to other aspects of behavioral modernity?

In addition to its cognitive properties, divergent thinking involves relaxed states of intuitive awareness that are also characterized by physical spontaneity, cheerful affect, playfulness, and sociability [40, 49, 50], which can be quantified in terms of personality characteristics. Personality refers to the way an individual learns to shape and adapt to an ever-changing internal and external environment [51].

Like divergent thinking, creative personality features are multi-faceted, including character traits (i.e., styles of rational self-government, with executive functions of self-directedness, legislative functions of cooperativeness, and judicial functions of self-transcendence) and temperament traits (i.e., emotional drives of curiosity about what is novel, willingness to take risks, willingness to work for social recognition, and perseverance for the sake of achievement) [36, 52, 53]. The two domains of temperament and character make it clear that a person’s potential for creativity cannot develop without both the wisdom to recognize what is valuable and the plasticity to adapt accordingly. The most widely used psychometric test for assessing both domains of the creative personality is the Temperament and Character Inventory (TCI) [51, 52, 54]. Tests of creative divergent thinking in verbal and pictorial tasks, creative personality traits as measured by the TCI, and direct assessments of lifetime creative achievements, are each highly reliable and validated by their strong correlations with one another even when general intelligence and demographic variables are controlled [47, 48]. Empirically, the TCI creative personality profile also measures human health in general, including physical, mental, and social well-being [19, 53, 55]. Put another way, the three domains of features of behavioral modernity identified by anthropologists are in fact interdependent aspects of modern human health and well-being. As a result, the TCI provides valid quantitative phenotypic measures with which to investigate the cognitive, emotional, and social functions, brain connectivity, and genetics underlying creativity, prosociality, and other aspects of well-being in modern humans in ways that are robustly replicable [19, 56].

Cognitive scientists have proposed that the creative ability of Sapiens to see the world and other people in new ways depends on several interrelated brain processes of learning and memory. Sapiens’ creativity is thought to depend on human brain functions for prospective learning (i.e., the encoding, storing, and retrieval of intended actions), constructive learning (i.e., recollection of the past and imagining the future), and the related capacities for theory of mind (i.e., the ability to attribute mental states to ourselves and others to facilitate empathic social interaction), the default mode (i.e., awareness of internal milieu without focus on external tasks), autobiographical memory (i.e., vivid recollection of past experiences with contextual awareness of when and where facts were learned), and story-telling (i.e., meaningful composition of narrative figural art and language), which are all aspects of self-awareness and share largely overlapping brain circuitry [57–59]. In turn, these processes underlying divergent thinking operate cooperatively with other processes underlying convergent thinking, which have complementary functions for problem-solving in successful daily living [39, 46]. Just as creative personality is multifaceted, its related brain functions are also multifaceted aspects of complex neurocognitive systems of adaptability that are measured by the TCI [19].

## How do complex adaptive abilities develop and evolve?

Complex adaptive traits become organized by developmental [60] and evolutionary [61–63] processes characterized by multi-finality (i.e., the same antecedents can have different outcomes, as in genotypic pleiotropy) and equi-finality (i.e., different antecedents can have the same outcome, as in heterogeneity from redundant genotypic paths), as we have investigated in detail for the genotypic–phenotypic architecture of the TCI [19, 64, 65]. These properties of complex systems are the basis for the important role of exaptation, that is when already occurring characters are co-opted to enable new adaptive functions [66]. Such plasticity permits the creativity to make new things out of old parts, or, more specifically, to produce complex adaptive phenotypes and genotypes via nonlinear dynamical interactions among constituent features to select for advantageous novel functions [23]. The development of such complex adaptive functions are likely to be positively selected in evolution when they are beneficial for survival and reproduction, as has been suggested for the evolution of creativity in Sapiens in response to unpredictable climatic fluctuations and resource variability that threatened survival [26], or when large and cooperative social groups and trade networks began to benefit from enhanced communication by language to facilitate communication [2, 7, 67].

As a result of the incompleteness of the archeological record, there has been substantial controversy about whether the features of behavioral modernity emerged as a full set all at once during the late Pleistocene [2, 5], or if some features emerged individually and/or successively and then became organized in progressive stages in Africa in response to increased environmental pressure after the common ancestor of Sapiens and Neanderthals had dispersed to Europe before 500 kya [15, 25, 68]. The most recent information suggests the adoption of more complex behaviors after 400 kya and the emergence of the most distinctive features of behavioral modernity after 100 kya [25, 26], which suggests the role of many genes in coordinated networks, as expected for such complex adaptive traits. The complex behaviors observed before and after 100 kya may be distinguished best by the difference between convergent and divergent thinking because the best documentation of the modern creative imagination is narrative figural art [2, 5, 32], which requires both symbolism and self-awareness. Evidence for such creative divergent thinking first appears after 50 kya, and the creative achievements of Sapiens continue to accumulate to this day [11].

In any case, the characteristics of behavioral modernity are certainly complex adaptive traits that cannot be understood by focusing on one brain function or one gene at a time. Unraveling the complexities of behavioral modernity presents many daunting challenges.

## Challenges of understanding the evolution of human creativity

Comparisons of Sapiens to other living anthropoid primates provide circumstantial evidence that changes in brain circuitry and related functions for symbolism and/or self-awareness account for the creative characteristics that distinguish behaviorally modern humans from other hominids [12, 45, 57]. However, cranial fossils provide only limited information about the brains of Neanderthals and other extinct hominids [12, 45, 69–71]. Archeological evidence indicates that Neanderthal cultures and technologies showed little of the spirit of innovation that animated their counterparts among the Sapiens who replaced them in Europe and western Asia beginning at some time over 40 thousand years ago [9]. Neanderthals had sophisticated executive skills and did produce the occasional expression that might be interpreted as symbolic, but all the artifacts suggesting this is dated after 130 kya [17] and mostly around 40 kya after Sapiens had begun to migrate out of Africa [72–75]. It is clear that the creative use of symbols by Neanderthals was not a routine part of their lives and cultures, and that, although undoubtedly complex, the relationship of the Neanderthals to the environment around them—and presumably also to each other—was profoundly different from the one that Sapiens exhibits today [5, 6, 9].

On the other hand, the Neanderthals were very close relatives of Sapiens, and undoubtedly shared some of their behavioral, emotional, and cognitive functions [1–3, 12, 45]. As expressed informally by the paleogeneticist Svante Pääbo, “I want to know what changed in fully modern humans, compared with Neanderthals, that made a difference. What made it possible for us to build up these enormous societies and spread around the globe, and develop the technology that I think no one can doubt is unique to humans? There has to be a genetic basis for that, and it is hiding somewhere in these lists [of nucleotide base pairs of human genomes] [76].”

Pääbo acknowledges that progress in answering this question has been limited for two major reasons: first, a large number of changes in the human genome after its divergence from the common ancestor of humans and chimpanzees 7–10 million years ago (mya), and second, the lack of knowledge of the functional consequences of these changes [1]. Progress has also been limited by a lack of knowledge of the complex genotypic–phenotypic architecture of traits related to human creativity and behavioral modernity: the genes that influence complex aspects of human personality, such as creativity, symbolism, prosociality, and language, are likely to involve many genes acting in coordinated networks, rather than independently [77]. In order to circumvent these problems, we began by characterizing the complex genotypic–phenotypic relationships that describe the architecture of modern human personality using the TCI [19, 64, 65].

## Genotypic–phenotypic relations underlying behavioral modernity

We evaluated modern human personality using the TCI because it provides highly reliable and empirically validated measures of creativity and other aspects of behavioral modernity that are heritable and neurobiologically grounded, including physical, emotional, social, cognitive, and spiritual aspects of well-being [19, 42, 47, 56, 78, 79] as well as, or better than, other available tests [54]. The TCI accounts for two domains of personality based on distinct forms of learning and memory: temperament (i.e., the unconscious component of personality—associatively conditioned habits and emotional reactivity) and character (i.e., the self-regulatory components of personality—what people make of themselves intentionally and/or creatively) [51]. It was developed as a comprehensive measure of human personality, and captures the characteristics of behavioral modernity, including creativity and prosocial behavior, as reviewed in the preceding section and elsewhere [19, 42, 47, 48, 54, 56, 78, 79]. The TCI indices of creativity and well-being also predict subjective and objective measures of physical health, including healthy longevity [19, 55, 80].

We used data-driven methods to conduct genome-wide association studies of the TCI in three different samples with different environments and cultures (Finns, Germans, and Koreans). In this way, we were able to deconstruct the complex genotypic–phenotypic networks and environmental interactions underlying modern human temperament and character [64, 65]. These methods properly account for the properties of complex adaptive systems, including pleiotropy and genetic heterogeneity [81] (also see “Methods”).

More specifically, we proceeded in steps to characterize phenotypic-genotypic relationships at multiple levels of organization. First, in an epidemiologically representative sample of 2149 Finns, we identified sets of single-nucleotide polymorphisms (SNPs) that naturally cluster within particular individuals regardless of phenotype. Second, we uncovered five clusters of people with distinct configurations of the 13 facets of the self-regulatory domain of human personality (i.e., the character dimensions of Self-directedness, Cooperativeness, and Self-Transcendence) regardless of genotype. Third, we found 42 SNP sets that were significantly associated with the character profiles and identified 727 gene loci. We replicated 95% of the 42 SNP sets in a sample of 902 healthy Germans and a sample of 1092 Koreans, as well as their association with the character clusters [64]. The character-associated genotypic sets were found to modulate specific molecular processes in the brain for intentional goal-setting, self-reflection, empathy, episodic learning and memory, and healthy longevity.

Remarkably 68% of the 727 genes associated with character were unique to a single character profile [64]. As a result, there were multiple groups of genes that led to each individual character trait. For example, high self-directedness occurred in different individuals by means of distinct molecular processes of the genotypic networks that depended on particular configurations of Self-directedness with other aspects of character traits. That is, the genes for Self-directedness were different in people with the creative character profile (i.e., all 3 character dimensions are high so valued goals are unselfish, prosocial, and altruistic), the organized profile (Self-directedness and Cooperativeness are high but Self-transcendence is low, allowing for both personally and socially responsive action for mutual benefit but not sacrifice for others), or the resourceful profile (only Self-directedness is high, leading to self-centered motives).

Next, we repeated this process with the 12 facets of human emotional drives (i.e., the temperament dimensions of Novelty Seeking, Harm Avoidance, Reward Dependence, and Persistence) [65]. We uncovered three clusters of people with distinct temperament profiles regardless of genotype. One cluster was specified by low Novelty Seeking, high Reward Dependence, and high Persistence, which we designated as the reliable temperament set, as discussed in detail elsewhere [56, 82]. The other temperament clusters were the antisocial cluster and the emotionally hypersensitive cluster. 51 SNP sets were significantly associated with temperament clusters. The 736 genes that mapped to these SNP sets were enriched in molecular pathways activated by associative conditioning in animals, including the ERK, PI3K, and PKC pathways that are crucial for the modulation of synaptic plasticity and long-term learning involved in associative conditioning of emotional reactivity, social attachment, and persistence. We replicated 90% of the 51 SNP sets for temperament clusters in the healthy German and Korean samples.

The genes we uncovered for temperament and character overlapped partially, so we evaluated the organization of the temperament and character clusters jointly. We uncovered three phenotypic networks that accounted for the joint relations of clusters of temperament traits with clusters of character traits. We designated these joint temperament-character clusters as the (i) emotional-unreliable network (i.e., people who were highly emotionally reactive with little self-control or creativity), (ii) organized-reliable network (i.e., people with strong self-control of emotional conflicts and goals, but little creativity), and (iii) creative-reliable network (i.e., people who were highly creative, prosocial, and insightful in appraisal of values and theories) [19]. We found that these phenotypic networks were nearly disjoint (i.e., shared few subjects or phenotypic features) (see Supplementary Fig. 1). Each of the three phenotypic networks was strongly correlated with a different multi-locus genotypic network (see Supplementary Figs. S2 and S3).

The functions of the genes that mapped to the genotypic networks were found to regulate distinct systems of learning and memory underlying personality: (i) a multi-locus network of 249 genes for regulation of emotional reactivity, associative conditioning, and social attachments, which we designated as the “emotional reactivity” network; (ii) a multi-locus network of 438 genes for regulation of intentional goal-seeking, such as purposeful acquisition of food, manufacture of tools, cooperative team-work, logical analysis, and symbolization, which we designated as the “self-control” network; and (iii) a genotypic network of 574 genes for episodic learning and autobiographic memory of a person’s life as a narrative with past, present, and future within which the person can explore alternative perspectives with intuitive insight and creative imagination, which we designated as the “self-awareness” network. It is remarkable that 73% of the 972 genes in these three networks are unique to a single network. It is rare to find such a strong separation of clusters specified by such complex sets of phenotypic and genotypic variables [19].

The genes we identified for temperament and character accounted for nearly all the heritability of personality expected from twin studies [64, 65]. The strong relations of the three temperament-character phenotypic networks to three major genotypic networks for human adaptability provided us with valuable tools for evaluating the evolution of human creativity and other aspects of behavioral modernity by comparing the genomes of chimpanzees (*Pan troglodytes*) and Neanderthals to those of modern humans.

## Hypotheses to be tested

We hypothesized that the three nearly disjoint genotypic networks for human adaptability evolved in successive steps during the evolution of modern human personality. To test this hypothesis, we studied the 972 genes identified for personality in Sapiens, many of which were also found in the genomes of Neanderthals and chimpanzees. While only distantly related, these are the two species closest to modern humans that have well-characterized genomes comparable to the high-coverage genomes of modern humans [1]. We hypothesized that the three networks differ from each other in Sapiens, Neanderthals, and chimpanzees. Specifically, we hypothesized that (i) chimpanzees would have genes only in the emotional reactivity network, (ii) both Neanderthals and Sapiens would share many genes for intentional self-control, which was already evident in their common human lineage, and (iii) genes found only in Sapiens would be most frequent in the network for creative self-awareness, as previously predicted on the basis of coincident changes in brain and behavior during hominoid evolution [12, 45]. Once we identified the genes that were unique to modern humans from these analyses, we evaluated what types of genes distinguished the three networks. We also evaluated alternative transmission patterns and environmental conditions that may account for the sudden emergence of creativity in modern humans. Finally, we examined where the genes for learning and personality that are unique to modern humans are expressed in the brain.

## Subjects and methods

### Modern human subjects

Our sample of Sapiens was the Young Finns Study, an epidemiological study of 2149 healthy Finnish subjects who were assessed in 1997, 2001, 2007, and 2012 [83]. All subjects had thorough standardized genotypic, environmental, and phenotypic assessments, including administration of the TCI [64, 65].

### Personality indicators of behavioral modernity, creativity, and well-being

The Finnish subjects completed the TCI with 240 self-reported items using a 5-point Likert scale [84]. The internal consistency of scales and their re-test reliability were strong: *r* > 0.8 for dimensions and >0.65 for individual subscales between follow-ups, including the 15-year follow-up. The averages of the scales and subscales scores across the four assessment occasions were utilized to reduce measurement error.

All subjects completed the TCI to assess four dimensions of temperament (Harm Avoidance, Novelty Seeking, Reward Dependence, and Persistence) and three dimensions of Character (Self-directedness, Cooperativeness, and Self-transcendence). Each of these dimensions has multiple facets (subscales) measuring the expression of that dimension in different situations. Descriptions of high and low scorers on each dimension and its subscales are presented in Supplementary Table S1.

Our prior data-driven analyses of the genotypic–phenotypic architecture of the TCI uncovered a naturally occurring hierarchical structure that is important for understanding the complex relations of phenotypic indicators of genotypic predispositions to human behavioral modernity that can be derived from the TCI, such as creativity, well-being, self-awareness, and self-control, as we have described in detail elsewhere [19, 82] and in Supplementary Information. Human personality can be described at three levels of complexity from (1) individual temperament and character dimensions, each composed of the sum of their subscales, as shown in Supplementary Table S1, (2) genetically independent multi-trait temperament profiles or multi-trait character profiles [64, 65], and (3) joint networks of temperament and character profiles that indicate integration of multiple learning processes from each of the three major systems of human learning and adaptability, as described elsewhere [19] and briefly in the introduction.

We used two indices of health derived from the TCI, an index of well-being and an index of resilience from ill-being. We have confirmed the validity of the creative personality profile in which all three TCI character traits (i.e., Self-directedness, Cooperativeness, and Self-Transcendence) are highly developed as an index of well-being in multiple samples of modern humans in different cultures [19, 52, 55, 80]. Likewise, ignoring Self-Transcendence, the sum of Self-directedness and Cooperativeness is an indicator of resilience from ill-being in many cultures [19, 52, 55, 80]. In our sample of Finns we confirmed the validity of these two indices with independent measures of positive affective balance, perceived social support, physical behaviors (exercise, smoking, diet), and objective laboratory findings for ideal health as recommended by the American Heart Association, as described elsewhere [19] and in Supplementary Table S2.

### Gene annotation

The study has been carried out with 972 genes mapped to the three phenotypic networks: Creative-Reliable, Organized-Reliable, and Emotional-Unreliable (Supplementary Fig. S1) [19]. We refer to the corresponding genotypic networks as the Self-awareness, Self-control, and Emotional Reactivity networks, respectively (Supplementary Table S1, and Figs. S2, S3). The annotations of individual genes were obtained using the perl API of Ensembl [85] versions 87-92 (Supplementary Table S3) and classified according to their biotype distinguishing between protein-coding genes, non-coding RNA genes, and pseudogenes (Supplementary Table S4).

### Comparative genomics

Chimpanzee orthologs for the 972 genes related to personality in modern *Homo sapiens* were obtained by accessing the CHIMP2.1.4 database, which uses the *Pan troglodytes* model (7/20/16) built from genome (v.2.1.4) with gene model files (R.89) from Ensembl using the Perl API [86]. The orthologous genes for other primates (Bonobo, Chimpanzee, Gibbon, Gorilla, Human, Macaque, Marmoset, and Orangutan,) were obtained using programmatic access to resources in Ensembl [87].

Neanderthal orthologs of the 972 genes related to personality in Sapiens were identified in annotated data of the Neanderthal Genome Project [88]. The replicability of these findings was then evaluated in separate analyses of the high-coverage genomes of the Altai Neanderthal [88], another Neanderthal from the Vindija cave [89] (specimen 33.19, http://cdna.eva.mpg.de/neandertal/Vindija), and a third from the Chagyrskaya cave [90] (http://cdna.eva.mpg.de/neandertal/Chagyrskaya). These analyses enabled us to identify genes that chimpanzees and/or Neanderthals shared with modern humans from those that were only found in modern humans (Supplementary Table S5) and then to compare their characteristics (Supplementary Tables S6 and S7).

### General statistical methods

We used the Analysis of Variance (ANOVA) to test the null hypothesis that the three studied networks are similar in terms of the genes that compose them within one species and across species (Sapiens, Chimpanzee, and Neanderthal). To do so, we utilized both the ANOVA for independent and correlated samples, one per network (Self-awareness vs Self-control vs Emotional Reactivity) in each of the species. Then we applied post-ANOVA comparisons using *Tukey’s range Honestly Significant Differences* (HSD) Test to evaluate the specific differences between pairs of networks (e.g., Creative vs Organized). We used the ANOVA test as implemented in Concepts & Applications of Inferential Statistics, Richard Lowry 1998–2021, http://vassarstats.net/anova1u.html, and in the rstatix package in R.

For clarity in reporting results, *p* values reported in ANOVA and *Tukey’s HSD test* descriptives were rounded up to more conservative significance values 0.0001 and 0.01, respectively, which indeed tend to exceed the E-30 and E-10 values, respectively. The ANOVA effect size was calculated as the *f* value defined by Cohen [91], where he proposed the following interpretation of this value: *f* = 0.1 is a small effect, *f* = 0.25 is a medium effect, and *f* = 0.4 is a large effect. All other parameters used in each measurement of ANOVA were calculated as usual [91, 92], and full summaries of all our ANOVAs are provided in Supplementary Information and Supplementary Tables S8–S12.

### Genotypic estimation of the behavioral modernity of Neanderthals

The number of individual genes that Neanderthals shared with modern humans may not be an adequate indicator of their impact on creativity and other aspects of modern human functioning. We, therefore, evaluated the impact of genes on the predisposition to modern human well-being as an indicator of behavioral modernity by estimating their relative roles in specific SNP sets to take into account the interactions among coordinated sets of genes that impact well-being.

In order to extract prototypical samples of humans with distinctive Neanderthal-like features and distinctive Sapiens-like features, we first identified the genes found only in Sapiens and the genes Neanderthals shared with Sapiens, excluding genes present in chimpanzees (Supplementary Table S3). Then we cross-correlated these genes with the original SNP sets in which they had been detected in relation to character [64] and/or temperament [65] (see Supplementary Information). We selected SNP sets found in the genotypic networks for self-awareness, self-control, and emotional reactivity, for which we already had measured the associated levels of functioning in modern humans, including two indices (well-being and resilience from ill-being) [19]. From the measures of well-being that we had for SNP sets that contained one or more of the genes that Neanderthals shared with Sapiens, we then estimated the mean well-being of Neanderthal-like humans by weighting the well-being of people in those individual SNP sets by the proportion of genes present in Neanderthals compared to Sapiens in that SNP set for each of the networks. Likewise, we estimated the mean well-being of prototypical Sapiens-like humans from the measures of well-being in SNP sets that contained one or more of the genes found only in modern humans. Finally, we compared the levels of weighted well-being in SNP sets from the Neanderthal-like humans to Sapiens-like human prototypes using ANOVA statistics, including post-ANOVA comparisons and effect sizes. Finally, we estimated the relative genotypic modernity of these prototypes for the two species from the ratio of their mean levels of well-being.

### Horizontal gene transfer (HGT)

In order to determine if genes mapped to the three phenotypic networks could have been horizontally acquired, we calculated their overlap to the regions of HGT identified by Huang et al. [88] in the human reference genome hg 19 [93].

### Derived allele frequency (DAF)

We compared the DAF scores for long-intronic-non-coding (linc) RNA genes (Supplementary Table S4) found in Neanderthals with those found only in Sapiens to test for differential selection (Supplementary Tables S6 and S7). DAF scores [94] were calculated for lincRNA genes, including their exons and promoters, using the AnnLoc tool (http://annolnc.cbi.pku.edu.cn). DAF scores of 0.1 or less are associated with reduced diversity indicative of purifying selection (i.e., negative selection against deleterious alleles), whereas DAF scores >0.1 indicate increased diversity, as may occur with nonfunctional alleles, positive selection of advantageous alleles, or addition of new advantageous alleles in genes by HGT [95, 96].

### Gene co-expression in brain

To evaluate the functions of the genes we found only in Sapiens further, we used Process Genes List to analyze lists of genes that mapped to particular SNP sets with at least one gene found only in Sapiens [97]. This machine learning method uses the Allen Brain Atlas to calculate a normalized average mRNA expression level in each brain region for lists of each gene set. Brain regions in which those genes were significantly co-expressed were identified and displayed in brain images.

Further details about all our methods and statistical analyses are available as Supplementary Information.

## Results

### Genotypic personality networks distinguish Sapiens from other hominoids

We first tested which of the 972 genes associated with joint temperament-character networks of Sapiens (Supplementary Table S3) were also present in genomes of Neanderthals and/or chimpanzees. We found 509 genes for modern human personality in all three hominoids, 148 in Neanderthals but not in chimpanzees, 48 in chimpanzees and not in Neanderthals, and 267 only in Sapiens (Table 1).

**Table 1:** Statistical analysis of the 972 genes for modern human personality by personality network and species: Neanderthals (N) and chimpanzees (C)*.

	# TotalGenes/species		# Genes limited to species			
	N	C	C & N	N, not C	C, Not N	Not C Not N
Genes	653	557	509	148	48	267
Self-awareness	372	320	287	85	33	169
Self-control	303	251	236	68	16	118
Emotional Reactivity	178	156	141	38	16	54
Genes/network						
Self-awareness	0.65	0.56	0.50	0.15	0.06	0.29
Self-control	0.69	0.58	0.53	0.15	0.04	0.27
Emotional Reactivity	0.71	0.63	0.55	0.15	0.06	0.22

*972 genes for personality and learning in modern humans included 574 in the self-awareness network, 438 in the self-control network, and 249 in the emotional reactivity network. Their distribution in chimpanzees (C) and Neanderthals (N) were examined by species overall and by network within species.

**Fig. 1:**
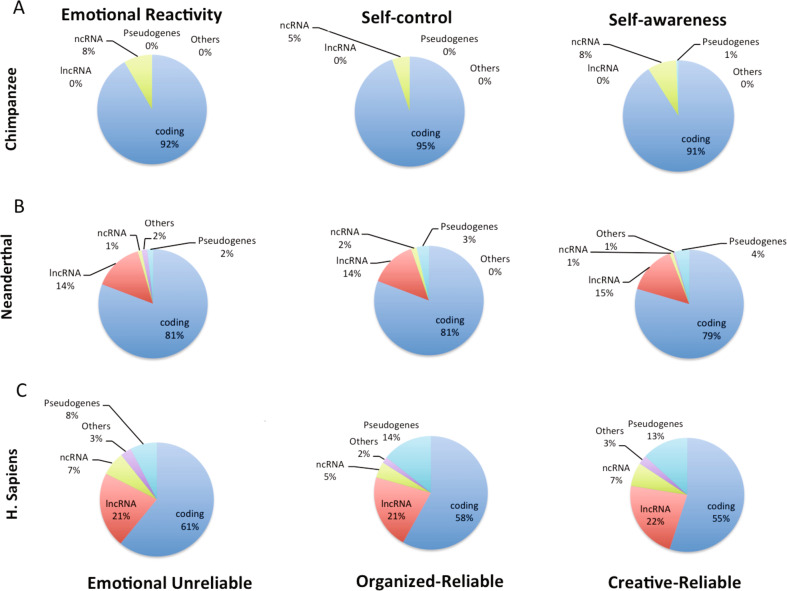
Comparison of types of genes in 3 hominoid species. Comparative analysis of the distinct types of genes (as defined in Supplementary Table S4) belonging to the Emotional reactivity, Self-control, and Self-awareness networks of genes present in (A) Chimpanzees (*Pan troglodytes*) (B) Neanderthals (*Homo neanderthalensis*) and (C) modern humans (*Homo sapiens*).

We hypothesized that the genes that mapped to the genotypic networks for emotional reactivity, self-control, and self-awareness would be differentially present in the genomes of chimpanzees, Neanderthals, and Sapiens. To test this, we performed ANOVA for the genes in the three networks along with contrasts of the possible pairs of species under assumptions of correlated samples (as occurs with vertical inheritance from parent to offspring) or of independent samples (as occurs with HGT from organisms other than parents), as summarized in Table 2 and described in Supplementary Table S8. We found that the three species differ significantly from one another for each of the three networks whether or not the samples are correlated (239 > *F (2[1664,2913])* > 16*, p* < 0.0001) (Table 2, Fig. 1, Supplementary Table S8). The differences among the species were large for genes related to self-awareness (Cohen’s effect size *f* = 0.46), intermediate for self-control (*f* = 0.35), and small for emotional reactivity (*f* = 0.21) (Supplementary Table S8).

**Table 2:** One-way analysis of variance (ANOVA, *p* < 0.0001) for the significance of the differences in the number of the 972 genes associated with personality in modern humans among the three species (ANOVA, *p* < 0.0001) with contrasts of numbers of these genes in pairs of the species depending on whether the samples are assumed to be correlated or independent. Significance of each comparison corrected for number of tests is shown. The species include modern *Homo sapiens* (“Sapiens”), *Homo neanderthalensis* (“Neanderthals”), and *Pan troglodytes* (Chimpanzees).

	Self-awareness	Self-control	Emotional reactivity
Correlated Samples			
Sapiens vs Chimpanzee	0.01	0.01	0.01
Sapiens vs Neanderthal	0.01	0.01	0.01
Chimpanzee vs Neanderthal	0.01	0.01	0.05
Independent Samples			
Sapiens vs Chimpanzee	0.01	0.01	0.01
Sapiens vs Neanderthal	0.01	0.01	0.01
Chimpanzee vs Neanderthal	0.05	0.05	Non-significant

In pair-wise comparisons, Sapiens differed significantly from Neanderthals as well as from chimpanzees in the genes found in each of the three networks, whether or not the samples are correlated (*Tukey’s HSD test*, *p* < 0.01) (Table 2, Supplementary Table S8). There was only a small difference between Neanderthals and chimpanzees in the genes they had in each of the three networks; the difference was weakly significant for each of the networks if the samples are considered correlated (*Tukey’s HSD test*, *p* < 0.05) and insignificant for the emotional reactivity network if the samples are independent (Table 2, Supplementary Table S8). Later we examine the possible occurrence of vertical and horizontal transfer in the evolution of human personality, but the differences observed here in Table 2 were about the same whether or not the samples are considered correlated; the one exception was that the smallest difference (i.e., between Neanderthals and chimpanzees in the genes regulating emotional reactivity) was not significant if the samples are considered independent. In sum, we found that the genes present in each of the three genotypic networks differ among each pair of the three hominoid species (*Tukey’s HSD test*, *p* < 0.01 to *p* < 0.05).

Next, we observed that the genes in the three networks are represented in hominoid species as cumulative additions, consistent with the hypothesis that Neanderthals were similar to chimpanzees in their genes for emotional reactivity but were intermediate between chimpanzees and Sapiens in the number of genes present in both the self-control and self-awareness networks (Fig. 1). Of the 972 genes significantly associated with personality in Sapiens, 653 were present in Neanderthals and 557 in chimpanzees (Table 1).

When compared to chimpanzees Neanderthals did not differ in their proportions of genes for emotional reactivity whether all genes related to personality in each species were considered (Table 1, e.g., 71% of 653, 62% of 557), or the total genes for human personality were accounted (18% vs 16% of 972, *F (1, 1972)* = 1.76, not significant) (Supplementary Table S9). Putting aside the 54 genes found only in modern humans, 72% of the 195 genes for emotional reactivity were common to all three species.

However, when compared to chimpanzees Neanderthals did have a greater proportion of the genes for self-control (32% vs 25% of 972, *F (1, 1942)* = 6.86, *p* < 0.008) and for self-awareness (38% vs 33% of 972, *F (1, 1942)* = 9.1*, p* < 0.0001) when all genes for human personality were considered (Supplementary Table S9).

Putting aside the genes for human personality present in chimpanzees, we found Neanderthals had only 33% of the genes in the self-awareness network of Sapiens (viz, 85 of 254 genes), 37% of the genes in self-control network (viz, 68 of 186), 41% of the genes in the emotional reactivity network (viz, 38 of 92). In other words, excluding the genes present in chimpanzees, 67% of genes for self-awareness, 63% of the genes for self-control, and 59% of the genes for emotional reactivity were found only in Sapiens.

Nevertheless, we recognized that the number of genes that Neanderthals shared with Sapiens might not be a direct indicator of their impact on creativity and other aspects of well-being because of the modular organization of genes in complex systems. Specifically, we needed to evaluate the impact of individual genes on well-being by estimating their relative roles in specific SNP sets to take into account the interactions among coordinated sets of genes that impact well-being.

We, therefore, estimated the impact on well-being of the genes shared by Neanderthals and Sapiens in comparison to the impact on well-being of the genes found only in Sapiens. In order to extract prototypical samples of humans with distinctive Neanderthal-like features and distinctive Sapiens-like features, we first identified the 267 genes found only in Sapiens and the 148 genes Neanderthals shared with Sapiens, excluding genes present in chimpanzees (Table 1, Supplementary Table S3). We estimated the mean level of well-being of Neanderthal-like humans from the well-being of individuals in naturally occurring clusters of genes (i.e., SNP sets) that included one or more of the 148 distinctive genes of Neanderthals. We estimated the mean level of well-being of prototypical Sapiens-like humans from the well-being of individuals in naturally occurring clusters of genes (i.e., SNP sets) including one or more found only in Sapiens.

We found that Neanderthal-like groups of genes enhanced well-being more than expected from the number of genes they shared with modern humans, but the mean levels of well-being were still consistently lower for Neanderthal-like humans than for Sapiens-like humans (Supplementary Table S10). The differences between these prototypical groups were similar in comparisons based on two indices of healthy functioning (well-being and resilience from ill-being) for each of the three genotypic networks, including self-awareness and self-control (*F (3,252)* = 34, *F (3,454)* = 35, *p* < 0.0001) and emotional reactivity (*F (3, 112)* = 15.5, *p* < 0.0005) (Supplementary Table S10). Specifically, the impact of genes for self-awareness on the well-being of Neanderthal-like humans was 70% of that in Sapiens, which is a rather large difference (effect size *f* = 0.34). Likewise, the combined impact of genes for self-control and self-awareness on the well-being of Neanderthal-like humans was 67% of that of Sapiens (effect size *f* = 0.28). The impact of genes for emotional reactivity on the well-being of Neanderthal-like humans was 61% of that of Sapiens (effect size *f* = 0.20). Similar findings were obtained indicating less resilience to ill-being in Neanderthal-like individuals also (Supplementary Table S10).

### Types of genes distinguish between personality networks and hominoid species

We analyzed the types of genes for the modern human temperament-character networks that are present in each hominoid group, as shown in Fig. 1 (see Supplementary Table S4 for type descriptions). Of the 557 genes present in chimpanzees, 92% were protein-coding and none were long-non-coding (lnc) RNAs or pseudogenes. Of the 653 genes present in Neanderthals, 81% were protein-coding and the rest were lncRNAs (14%), pseudogenes (2%), and non-coding (nc) RNAs (1%). Of the 972 genes associated with personality in Sapiens, only 61% were protein-coding and there were many lncRNAs (21%) and pseudogenes (8%). These distributions varied little across the networks within each hominoid group, as expected from our hypothesis that there were successive incremental steps between species to enhance regulation of the coordinated expression of groups of genes within each species (Fig. 1).

**Fig. 2:**
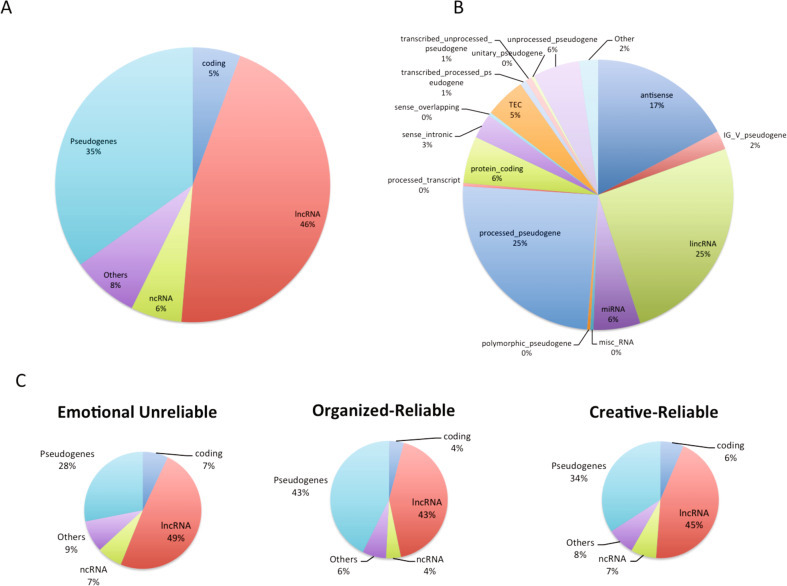
Types of genes found only in modern humans. Comparative analysis of the distinct types of genes (as defined in Supplementary Table S4) found exclusively in modern *Homo sapiens*: **A** Broad gene categories, **B** Fine-grained gene categories, and **C** Analysis of the genes from **A** in the genotypic networks associated with the Emotional-unreliable, Organized-reliable, and Creative-reliable phenotypic networks of modern humans.

The presence of lncRNAs and pseudogenes strongly distinguished the types of genes found in the three personality networks of humans (Neanderthals and Sapiens) from those found in chimpanzees (*F (5,1926)* = 91.1, *p* < 0.0001, effect size *f* = 0.69, and *Tukey’s HSD test*, *p* < 1E-23, Fig. 1 and Supplementary Table S11). Chimpanzees had none of the lncRNAs associated with modern human personality (Fig. 1). Sapiens had more lncRNAs than Neanderthals (21–22% vs 14–15%, *Tukey’s HSD test*, *p* < 1E-12) (Fig. 1). The differences in candidate regulatory genes among the hominoid groups were confirmed with the genes present only in Sapiens (Fig. 2).

### Distinct type of genome evolution and direction of selection in Sapiens

We tested for the presence of ancestral genes involved in human personality by searching for orthologs in 57 organisms belonging to the following taxonomic groups: Primates, other Mammals, Marsupials, Monotremes, Avians, Reptiles, Amphibians, Fish, Cyclostomes, Tunicates, Insects, and Nematodes (Supplementary Table S1, Supplementary Fig. S4A). We found that 557 of the 972 genes related to personality have orthologs in these species, suggesting inheritance through common ancestry. The remaining 415 genes apparently without known orthologs might have been acquired independently. Independent transmission might have occurred, for example, as a result of HGT, which is widely implicated in the human genome, particularly in primates [88]. We found that 39 genes associated with human personality were in previously known HGT regions (Supplementary Table S3). Thus personality-related genes are enriched in known HGT regions (4.0% of 972) compared to overall rate in the human genome (1.1%, 642 of 57,905) [88].

About 65% of the 415 genes without orthologs belong to the self-awareness network, which is strongly associated with the creative-reliable personality profile. In contrast, we found that the personality-related genes located in HGT regions were enriched in all three genotypic networks with slightly higher rates for genes in the self-control network (5.5%, 24 of 438 genes) and emotional reactivity network (4.0%, 10 of 249) than in the self-awareness network (3.0%, 17 of 574). Only two of the genes in HGT regions were found only in Sapiens, suggesting HGT had little role in the emergence of behavioral modernity.

In contrast, our findings of lincRNAs provided evidence supporting a major role in the emergence of behavioral modernity. Among the 972 genes associated with personality in Sapiens, we found that 127 were lincRNAs without orthologs in 57 species (see Supplementary Information): 68 were present only in Sapiens, 59 in Neanderthals, and none in chimpanzees (Supplementary Table S2). Information about DAF scores was available for 60 lincRNAs unique to Sapiens (Supplementary Table S6) and 53 that are present in Neanderthals (Supplementary Table S7), enabling us to compare them (Supplementary Table S12). Among the lincRNAs unique to Sapiens (Supplementary Fig. S4C), those with DAF > 0.1 are more frequent than those with lower DAF for both their promoters (*F (1,110)* *=* 30.23*, p* < 0.0001) and their exons (*F (1,110)* = 9.78, *p* < 0.0022). Likewise, both promoters (*F* *(1,92)* = 45.35, *p* < 0.0001) and exons (F *(1,94) =* 11.75, *p* < 0.0019) are primarily under positive selection in lincRNAs that are also present in Neanderthals. However, lincRNA promoters in Sapiens, but not in Neanderthals, have DAF > 0.1 slightly more often than their exons (*F (1,102)* = 4.54, *p* < 0.03), suggesting that positive selection is acting on regulatory functions in Sapiens. Significantly, ~70% of the 49 lincRNAs unique to Sapiens and under positive selection (DAF > 0.1) were in the self-awareness genotypic network.

### Expression of gene sets unique to moderns in specific brain regions

94% of the 267 genes unique to humans were non-protein-coding genes, and 64% were associated with the self-awareness genotypic network (Supplementary Table S5). Their specific functions are largely uncertain except that in general they are suggested to coordinate complex processes of adaptation, plasticity, and health by regulating the co-expression of groups of other genes. Therefore, we evaluated the co-expression in different brain regions of the sets of genes that mapped to the same personality-related SNP set that contained at least one gene found only in Sapiens (Supplementary methods and Table S13). We calculated the average mRNA expression level in specific brain regions of multi-genic clusters related to character or temperament.

**Fig. 3:**
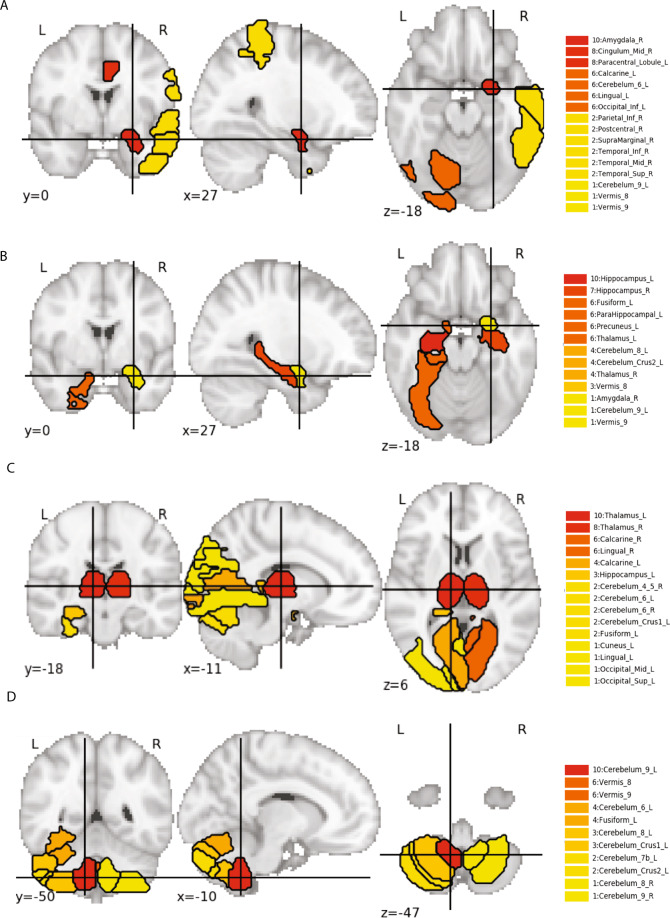
Brain regions in which genes found only in modern humans are overexpressed. Normalized average gene expression in Allen Human Brain Atlas in particular regions of the human brain in which there is significant co-expression of the constituent genes of SNP sets in which there is at least one gene found only modern humans. The highlighted regions are called regions of interest (ROIs) because they are the regions in which a given list of genes are significantly co-expressed when compared to the rest of the Allen Human Brain Atlas. The color code indicates the importance of the region, with red as maximum. **A** and **B** depict ROIs in which constituent genes of the Character SNP sets of the self-awareness network are highly expressed, whereas **C** and **D** depict ROIs in which constituent genes of the Temperament SNP sets of the self-awareness network are highly expressed.

The brain regions in which the identified multi-genic clusters unique to Sapiens were significantly over-expressed are displayed in Fig. 3. We confirmed the hypothesis that the genes related to the character of Sapiens were over-expressed in brain regions that have been involved in human self-awareness and autobiographical memory in prior functional brain imaging studies. Specifically, they were significantly over-expressed in late-myelinating regions of neocortex in frontal, temporal, and parietal regions (Fig. 3A), as well as in the associated areas of the thalamus, basal ganglia, cerebellum, and brainstem involved in cerebellar-thalamo-cortical, cortico-ponto-cerebellar, and cortico-striato-cortical loops important for intuitive insight and evaluation, which is automatic without deliberate analysis [88] (Fig. 3B, Supplementary Table S14). The pontine nuclei, the main source of input from the frontotemporal cortex to the cerebellum, are in the brain region with the densest co-expression of genes for both character and temperament (Supplementary Table S14).

In addition, we found that the genes related to the temperament were significantly over-expressed in the hippocampus, septum, amygdala, cingulate cortex, parahippocampal gyrus, fusiform gyrus, thalamus, cerebellum, and brainstem (Fig. 3C, D, Supplementary Table S14), as expected from the role of these regions in recognition, evaluation, and self-regulation of emotional expression.

When associated with genes found only in Sapiens, genes from both the self-awareness and self-control networks were significantly over-expressed in the brain regions that comprise the pathways for self-awareness and self-regulation of emotions and goals. Put another way, the regulation of gene co-expression by genes found only in Sapiens provided a mechanism to integrate self-awareness and self-control so that human emotions, goals, and values could be self-directed in ways that are coherent, reasonable, and advantageous.

## Discussion

This is the first study to identify the genotypic differences among chimpanzees, Neanderthals, and modern humans that may account for the rapid emergence of human creativity and other components of behavioral modernity, including its physical, emotional, cognitive, social, and spiritual features. In preparatory work we identified three naturally occurring genotypic networks for emotional reactivity, intentional self-control, and self-awareness. The 972 genes in these networks account for nearly all the heritable variation of human personality, including the characteristics of behavioral modernity (namely, creativity, prosocial behavior, and healthy longevity). Now we have found that 267 of these genes are absent in both chimpanzees and Neanderthal genomes, and we replicated this finding in three high-coverage Neanderthal genomes.

We also found that Neanderthals had nearly the same proportions of genes for emotional reactivity as chimpanzees. Excluding 54 genes found only in Sapiens, 72% of the 195 genes for emotional reactivity were common to all three species. On the other hand, Neanderthals were intermediate to chimpanzees and Sapiens in their proportions of genes for self-control and for self-awareness. Putting aside the genes for personality present in chimpanzees, Neanderthals had 33% of the genes for self-awareness and 37% of the genes for self-control that are present in Sapiens. Nevertheless, when we took into account the modular organization of these genes in clusters with other genes, we estimated the relative well-being of Neanderthal-like humans was 61–70% of that of prototypical Sapiens who carried genes found only in modern humans. Prototypical Sapiens have much stronger genotypic predisposition to the characteristics of behavioral modernity than Neanderthal-like humans, particularly from sets of genes in the self-awareness network associated with creativity, prosocial behavior, and longevity (*F (3,252)*, *p* < 00001, Cohen’s effect size *f* = 0.34).

In addition, we obtained evidence that the genes found only in Sapiens were likely to be regulatory and advantageous. Specifically, 94% of the 267 genes found only in Sapiens were not protein-coding, including many lncRNAs (46%), pseudogenes (35%), and ncRNAs (6%). 64% of the genes found only in Sapiens were in the self-awareness network, especially lncRNAs that we found to be under positive selection.

Finally, we tested the importance of the genes unique to Sapiens for human well-being and behavioral modernity by identifying the brain regions in which they were over-expressed. We confirmed that naturally occurring clusters of genes associated with one or more genes found only in Sapiens were over-expressed in the core brain regions for human self-awareness, which is strongly associated with the human well-being, including the characteristics identified by anthropologists as distinguishing Sapiens from other hominids whom they replaced by 40 kya.

With these key findings in mind, we will discuss both the anthropological and the genetic data available to test our hypotheses related to the successive emergence of nearly disjoint networks for regulation of emotional reactivity, intentional self-control, and creative self-awareness in the hominoid lineage of modern humans. From our preparatory studies of the phenotypi–genotypic architecture of human personality, we recognize that these three networks function cooperatively so that a person can learn to integrate their habits, goals, and values in adapting to changes in their internal and external milieu. Available information about the coincident changes in brain and behavioral functioning in the phylogeny of Sapiens help to guide our interpretation of our findings based on comparison of the genomes of chimpanzees, Neanderthals, and Sapiens.

### Emergence of the network for regulation of social emotions

The mammalian ancestors of anthropoid primates were mostly small, nocturnal, and solitary; but as temperatures cooled and tropical forests receded during the late Eocene, around 40 million years ago (mya), there was probably a selective advantage in social cooperation among higher primates as a protection against predators when foraging in the daytime [12, 14]. Social learning similar in kind to that of humans consequently developed among monkeys and apes, resulting in social attachment [98, 99] and the regulation of emotional reactivity based on social context and the reduction of emotional distress by reconciliation [100], as among chimpanzees today who, following a fight, often engage in mouth-to-mouth kissing and ventral embraces. Social learning also allows proto-cultural transmission of traditions in grooming, courting, foraging, and food preparation [101–103]. Emotional gestures and vocal calls facilitate social relations among triads and larger groups of higher primates, so that a third party, such as a high-ranking group leader, can intervene to resolve conflicts [100, 104].

On the other hand, while chimpanzees show emotional reactivity and learning abilities similar to those of a 2- or 3-year-old modern human child, they do not exhibit the regulatory capacities of older modern human children [105]. Chimpanzees use tools to solve simple tasks, like cracking nuts or catching termites; but they do not teach each other to manufacture and use these tools [1]. They can be taught to use signs and form two-to-four-word sentences at a rate consistent with behavioral conditioning, but, unlike modern human children, they do not spontaneously acquire symbolic language [45, 106, 107]. The self-aware memory of modern human children begins to mature around 4 years of age, and afterward they show greater capacity than chimpanzees for delay of gratification, reasoning about beliefs, and solving problems about internal memories [57, 105–108].

When the brains of higher primates are compared to those of more distant relatives of humans [12, 45], the prefrontal cortex is typically enlarged, projecting directly to the hypothalamus, striatum, thalamus, septum, and basal amygdala. Affective information is also relayed to the middle insular cortex, which allows regulation of sensuality. The mirror neuron system emerges, allowing the understanding of action and the imitation of observed behaviors, a necessary precursor of language. In great apes, there is also differentiation of the anterior insular cortex, allowing the enhanced emotional awareness that supports the communication of social emotions. On the basis of these findings of coincident changes in brain and behavior, we hypothesized that the genome of chimpanzees is likely to have the genetic network for regulation of emotional reactivity, but not those for either intentional self-control or creative self-awareness [12, 45]. Our current findings strongly confirm this hypothesis: the emotional reactivity network is well-developed in all three hominoid species that we evaluated. Putting aside the 54 genes found only in Sapiens, 72% of the 195 genes in the emotional reactivity network were shared by all three species (Table 1).

### Emergence of the network for regulation of intentional self-control

Early hominins rapidly became distinguished from great apes by a greater facility for purposeful goal-seeking behaviors such as tool-making and coordinated hunting for food [12, 45]. Current indications are that the use and manufacture of stone tools were introduced by archaically-proportioned “australopiths” (e.g., [109]) at a time when open habitats were becoming more widespread as tropical forests shrank. Subsequently, the possession of more or less modern limb proportions by the earliest properly diagnosable members of the genus *Homo* indicates that hominins had finally committed themselves to those open habitats by a little under 2 million years ago. This crucial transition is poorly documented in behavioral terms, but it certainly represented an extreme environmental and economic shift that must have had profound cognitive and social sequelae.

Once committed to open habitats, the brain size of hominins began to increase rapidly. *Homo ergaster* (literally, working man) was reasonably tall and slenderly built in the basic manner of modern humans, and introduced the Acheulian tool industry of symmetrical bifacial hand-axes before 1.6 mya. These implements were intentionally flaked to conform to a template held in their makers’ minds. Later hominines continued this tool-making tradition without radical innovation until around 400 kya [9, 10]. This archeological record of technological stasis for over a million years documents that early humans had the capacity for intentional self-control, but that humans living prior to 400 kya, including the common ancestor of Neanderthals and Sapiens, did not manifest the creativity associated with the genotypic network for self-awareness of Sapiens [12].

*Homo neanderthalensis*, a species that evolved from an endemic European precursor some 200 thousand years ago, was one highly evolved end-product of the human commitment to living in open habitats. Neanderthals were clearly purposeful and resourceful creatures who typically lived in small bands of perhaps 12–25 individuals that foraged across vast landscapes [110]. They were clearly sophisticated beings who were highly opportunistic in the resources they exploited: they hunted some frighteningly large prey when circumstances dictated (thereby possibly accounting for a reported high incidence of bone fractures [110]); at least occasionally they built shelters, and they controlled fire in hearths [111–113]. There is evidence at Shanidar cave in northern Iraq of a Neanderthal surviving to advanced age despite being severely handicapped by a useless arm, suggesting social cooperation and empathy for others within their small groups [113]. On the other hand, while Neanderthals buried their dead, they typically did so without the grave artifacts characteristic of later Cro-Magnon burials [113, 114]. Neanderthals produced artifacts that have been interpreted as symbolic art, but these infrequent expressions were simple and two-dimensional [73–75], possibly comparable to pictures produced by modern human children before the age of 7 years [115]. Their low genetic diversity suggests that they lived in small isolates with limited mating between groups [110, 116], although there is some evidence for female exogamy [115].

In the period following 40 thousand years ago the Neanderthals were rapidly replaced in Europe, albeit with some minor gene exchange [117], by invading *Homo sapiens* whose lives showed unprecedented cultural and technological sophistication. While still itinerant hunter-gatherers, these anatomically and behaviorally distinctive new humans populated the landscape in higher densities and brought with them the symbolic tradition of narrative cave art with use of pictorial depth cues in integrated compositions of great complexity and beauty [118]. This innovative practice of creating pictures from the imagination—“the mind’s eye”—is the most powerful indicator we have of the awakening of the modern sensibility, with its profusion of abstract but clearly meaning-laden signs in addition to the sophisticated animal images famous from such localities as Chauvet and Lascaux [73].

The brains of extinct humans are available only as fossil endocasts, limiting the observations that can be made. Compared to chimpanzees, fossil data document the emergence of hemispheric asymmetry along with bipedality in australopiths and non-Sapiens. Arising late in hominin history, Neanderthals had large brains that averaged about 1500 ml in volume, more or less identical to those of contemporaneous Pleistocene *Homo sapiens* (although modern human brains are almost 13% smaller [117]). However, those brains appear to have been organized differently from modern ones: Neanderthals had relatively larger visual areas, while Sapiens have expanded parietal lobes [69] and higher prefrontal regions. On the basis of these findings of coincident differences in brain and behavior, we hypothesized that the genome of Neanderthals would likely be found to have the genetic network for regulation of emotional reactivity and some of the genes of the network for intentional self-control, but not that for self-awareness [12, 45].

Our current findings confirm that the genotypic network for intentional self-control is well-developed in Neanderthals but not in chimpanzees. They also suggest that Neanderthals had acquired genes for self-control and self-awareness in numbers intermediate between modern human and chimpanzees. Excluding genes already present in chimpanzees, Neanderthals had 33% of the 254 genes for self-awareness and 37% of the 186 genes for self-control that are present in Sapiens. Taking into account the modular organization of groups of genes within human learning networks, we estimated that the relative level of genotypic predisposition to well-being and modernity of Neanderthal-like humans was 61–70% of that of prototypical Sapiens. When compared to prototypical Sapiens, the genotypic predisposition to modernity of Neanderthal-like humans is lowest for self-awareness (Cohen’s effect size *f* = 0.34). These findings suggest that the crucial event that sparked the emergence of behavioral modernity was the advanced evolution of the genotypic network for self-awareness in Sapiens, but we need to consider alternative explanations for these findings.

Of course, one possible alternative explanation is that all the genes present in Neanderthals may not have been documented in the genomic information currently available to us, even though we replicated our findings using the 2010 draft genome separately in each of the three high-coverage Neanderthal genomes that are available: Vindija 33.19 from the central range of Neanderthals in Croatia, as well as the genomes of a Neanderthal from the Altai Mountains and another from the Chagyrskaya Cave in Russia [110, 116, 117, 119]. These replicated findings provided robust support for our comparative analyses, but we still needed to know whether the genes we did find provided a mechanism that might account for the emergence of creativity.

### Emergence of the network for creative self-awareness

What mechanism promoted the emergence of the genetic network for creative self-awareness in behaviorally modern human beings? The brains of Sapiens are unique in having a system for self-awareness that connects the late-myelinating regions of the frontal, parietal, and temporal cortices [57, 120]. These most recently evolved regions of the brain are the final association areas in which information is integrated and evaluated, and are linked into a unified network for episodic memory by projections from visual cortex [12, 45]. Autobiographical learning and memory mediate awareness of the self as a continuous identity across space and time. Psychologically, the creative network is so-named because it is found in people who are imaginative, inventive, prosocial, and spiritual [42, 47, 48, 55, 80, 121]. Such self-transcendent thinking involves the ability to perceive oneself as a local aspect of a larger spatio-temporal whole, which permits thinking that is free and creative (i.e., “outside the box” of logical deduction and cultural tradition) and theoretically inductive (i.e., extrapolation beyond prior examples based on insight and creative imagination), as expressed in art, science, spirituality, and narrative syntactical language [12, 45]. On the basis of findings of the unique association of coincident changes in brain with cognitive functions for self-awareness and creativity, we hypothesized that only Sapiens were likely to have the genotypic network for self-awareness.

However, this hypothesis was only partially supported. We found that Neanderthals had only 33% of the genes for self-awareness present in Sapiens; but these genes, when organized in clusters with other human genes, were sufficient for Neanderthal-like humans to function at 61–70% of the level of well-being of prototypical Sapiens. This still does not inform us whether Neanderthals had crossed the genotypic threshold needed to have the potential to express some or all of the features of behavioral modernity, even if that capacity has not been adequately documented in the archeological record.

Therefore, we asked whether the genetic differences between Neanderthals and Sapiens revealed molecular mechanisms that qualitatively distinguished them and/or accounted for greater reproductive fitness in Sapiens. We found that the lincRNAs unique to Sapiens are under positive selection and are functionally different than those found in the Neanderthal genome. LincRNAs are known to evolve rapidly [122], and to influence complex patterns of adaptive functioning, plasticity, and health by regulation of gene expression [123, 124] and co-expression of groups of genes [125]. We found that 70% of the lincRNAs under positive selection and unique to Sapiens are in the genotypic network for self-awareness. When reared under conditions of parental warmth and tolerance, Sapiens with the genotypic network for self-awareness are likely to develop a creative-reliable personality profile characterized by creativity, altruism, and healthy longevity [19], thereby creating a distinctive social dynamic. This interpretation is directly supported by our additional finding that the genes for Sapiens are found in multi-locus genotypic clusters that are over-expressed in the brain regions that define the self-awareness network.

Furthermore, the characteristics of altruism and healthy longevity may have provided conditions necessary for kin selection for creativity in Sapiens as an adaptive response to intense ecological pressure from climatic fluctuations and unpredictable variability in resource availability in East Africa, but not Neanderthals who were not under the same pressures in Europe. The importance of prosocial environments for creative achievement is still evident in behavioral differences among modern humans observed today: even Sapiens with the genotypic network for self-awareness are still vulnerable to physical, emotional, cognitive, and social ill-being under hostile or inequitable social conditions [19], as shown in Figure S5D. Consequently, altruistic and creative behaviors are frequent, but inconsistent, features of Sapiens [121, 126].

Considering all the evidence available, we know that Neanderthals were intermediate between chimpanzees and Sapiens in the development of the genotypic network for self-awareness. We also know that Sapiens have a distinctive set of genes that are mostly in the self-awareness network, are under positive selection, and are not present in Neanderthals. Our genotypic findings document molecular mechanisms that may provide a likely explanation for the archeological record that has found only rudimentary evidence of creativity and other signs of behavioral modernity in Neanderthals. We, therefore, need to carefully consider these potentially crucial mechanisms in detail.

### Hypotheses about selection for creativity

The newly emergent creativity may have provided selective advantages to behaviorally modern humans beyond its purely cognitive advantages. Physiologically, it is associated with enhanced memory functions, health, and well-being (Supplementary Figs. 5 and 6), including a predisposition to longevity and resilience against stress, injury, and chronic diseases including cardiovascular and neurodegenerative diseases [19, 64, 65]. Living longer and healthier lives may have allowed behaviorally modern *Homo sapiens* to disperse rapidly and widely around the world, and it may also have helped individuals support their children, grandchildren, and others in interconnected social communities, thereby possibly leading to positive selection for traits such as creativity, innovativeness, prosociality, and wisdom [127–131]. We hypothesized that the genetic network for creativity was positively selected because we had previously found that longevity and well-being are promoted by the integration of creative functioning, plasticity, and virtues like moderation, altruism, and wisdom [19]. This hypothesis is further supported by our finding that 70% of the advantageous lncRNAs unique to Sapiens were in the self-awareness network, which is strongly associated with creativity, prosociality, and healthy longevity [19, 55, 80]. Hence it is a crucial observation that most of the key regulatory genes for creative self-awareness are only present in Sapiens, and not in Neanderthals: of the 130 lncRNAs in the self-awareness network, none were present in chimpanzees, 42% were shared by Sapiens and Neanderthals, and 58% were found only in modern humans (Table 1, Fig. 2, Supplementary Table S3).

### Role of LncRNAS in rapid evolutionary change

What mechanism can account for the rapidity of the evolution of creativity, healthy longevity, and fitness in Sapiens [1–4, 132]? Changes in mutation rates do not provide an explanation because they remained stable in the transition from archaic to modern humans [119, 133]. We considered mechanisms by which new genes appear in ways that do not depend on the mutation rate of ancestral genes [134]. We observed that 67% of the genes associated with human self-regulation and creativity were regulatory genes [64], including a significant predominance of lncRNA genes and pseudogenes when compared to the genes related to behavioral conditioning of temperament [65]. We know that differences in complexity of functions between species usually depend on differences in the regulation of gene expression of a highly conserved core of protein-coding genes, as has been shown for the differences between chimpanzees and humans [135–137].

More specifically, we know that lncRNA gene are often important regulators of gene expression [138] and are often acquired by horizontal gene transfer (HGT) [88]. HGT (i.e., the acquisition of genes from an organism other than a direct ancestor) allows genomes to expand rapidly, assemble new pathways, and express new functions [139]. HGT is the main mechanism for acquisition of new genes in prokaryotes and single-celled eukaryotes, and also is widespread in primates, including humans. Many new genes have been acquired throughout the modern human genome, especially protein-coding and lncRNA (e.g., lincRNA and antisense) genes [88]. Therefore, we tested the hypothesis that modern human beings acquired the genes that enabled the rapid evolution of creativity and healthy longevity by HGT. We found that genes for human personality are enriched in HGT regions, but the enrichment was observed for genes in the emotional reactivity network as well as the others. Furthermore, only 2 of the 39 genes we found in HGT regions were unique to modern humans. Therefore, we concluded that HGT may have contributed to personality development in hominoids in general, but it did not have a major role in the development of the creative personality or self-awareness.

In contrast, our findings that 70% of lincRNAs unique to humans and under positive selection were found exclusively in the self-awareness network does provide evidence of their involvement in the evolution of self-awareness and the various aspects of human well-being and behavioral modernity. Likewise 35% of the genes unique to Sapiens were pseudogenes, which are also often under positive selection in primates [140, 141] and involved in regulation of human cognition [142]. Pseudogenes were more frequent in genes associated with personality in Sapiens (8% of 972) than in Neanderthals (2% of 652). However, in Sapiens, pseudogenes were more frequent in the network for self-control (43%) than for self-awareness (28%). Therefore, lncRNAs appear to have played a more direct role in the emergence of creativity in Sapiens, although pseudogenes also contribute substantially to the differences between the two human species that emerged under distinct ecological conditions.

In contrast to the differences that we observed in biotypes between species, we found that the biotypes of the genes are similar for each of the three networks within each species (Fig. 1). In sum, both the differences in biotypes between species and the similarity of biotypes across adaptive networks within species support our hypothesis that the nearly disjoint genotypic networks are likely to have emerged in incremental steps. The initial emergence of intentional goal-setting in early hominins and later the emergence of the creative imagination of Sapiens has allowed modern humans to adapt to social and environmental challenges by brain functions that are associated with distinctive molecular processes and many regulatory genes that are found in modern humans, but not chimpanzees or Neanderthals.

### Strengths and limitations

The major innovation and strength of our study of the evolution of human creativity is our having begun by first characterizing the complex genotypic–phenotypic architecture of human personality that underlies the human capacity for self-awareness, symbolism, and creativity. We identified and replicated the genotypic networks underlying the three major systems for learning in Sapiens (behavioral conditioning, intentionality, and self-awareness). This allowed us to focus comparative genomic analyses on 972 genes that account for modern human personality and learning capacities.

A major challenge was that there is less information about the Neanderthal genome than there is for modern *Homo sapiens* and chimpanzees. The annotated genome from the Neanderthal Genome Project from 2010 is based on low-coverage data, nearly all of which was from the Vindija Cave in Croatia that lay in the central range of Neanderthals throughout most of their existence. Fortunately, we were able to replicate our initial findings with the complete high-coverage (~50×) genome of the Altai Neanderthal, which confirmed the same 267 genes of Sapiens that were absent in Neanderthals from Vindija. Our findings were also confirmed in separate analyses of two other high-coverage (~30×) genomes from caves in both Croatia and Russia, so our findings are robust.

Another limitation of all work about complex phenotypes is that extinct hominids can never be available for quantitative phenotypic assessments comparable to those of modern humans using the TCI. Fortunately, the TCI has been directly validated with measures that correspond to descriptions of behavioral modernity by paleoanthropologists. Our genotypic measures and phenotypic measures are strongly related (Supplementary Fig. S3 and Table S2), and we have characterized the complex hierarchical and modular organization of their phenotypic–genotypic relations. As a result, we were able to use our genotypic measures to estimate the relative genotypic predisposition to the well-being and modernity of Neanderthal-like humans to prototypical Sapiens. Unfortunately, we still cannot state definitely what aspects of self-awareness Neanderthals may have displayed. We know that even chimpanzees have some rudimentary aspects of self-awareness, including mirror recognition and some recognition of self-agency [143]. However, chimpanzees lack flexibility in reasoning about abstractions, such as beliefs and intentions, an aspect of creativity and self-awareness that emerges between 3 and 5 years of age in modern human children [108, 144]. Therefore, we expect that Neanderthals had at least rudimentary aspects of self-awareness intermediate between chimpanzees and Sapiens, even though Neanderthals lacked most of the lncRNAs for self-awareness that we found in modern humans.

Because we focused only on the 972 genes that account for personality in Sapiens, we cannot exclude the possibility that Neanderthals had genes that were not present in Sapiens and influenced their personality and learning abilities. These genes could have been inherited from the common ancestor of Neanderthals and Sapiens or acquired by Neanderthals subsequently. Any such unique Neanderthal genes could have had functions homologous or distinct to those present in modern humans. However, we have identified what genes found in Sapiens, but not in Neanderthals, account for the emergence of the advantageous capacities of Sapiens, including creative self-awareness, prosocial behavior, and healthy longevity. Available behavioral data also indicate that these same capacities were absent in Neanderthals and other extinct hominids, and more detailed genotypic-phenotypic analyses comparable to what we have done in modern humans are impossible. Therefore, it is likely to be much more useful to pursue a more detailed understanding of the functions of the genes unique to Sapiens than those unique to extinct hominids.

Another major challenge was the limited information known about the functions of the non-coding RNA genes that comprised most of the genes found only in Sapiens. Fortunately, lncRNA genes have been shown to regulate the expression of sets of other genes, so we were able to identify the specific brain regions in which the multi-locus genotypes that map to the SNP sets related to self-awareness in Sapiens are expressed. Our findings of gene expression in the brain of the self-awareness network confirmed findings from functional brain imaging about the brain regions involved in various functions of self-awareness, including autobiographical memory, prospection, theory of mind, and the default mode [59]. Our findings extended this by revealing additional subcortical structures that are involved in cortical feedback loops important for the automatic processing and integration of information in self-awareness. The replicability of our genetic findings and their meaningful association with specific brain circuitry for complex human functions provides strong evidence for the validity of the data-driven methods we have developed and applied to characterize complex adaptive systems [64].

### Overview

Our findings have broad implications for understanding what enabled Sapiens to displace Neanderthals and other species of *Homo* in the geologically recent past, as well as literally to reshape the world during the Anthropocene. Living longer, healthier lives may have promoted and valorized the extended periods of juvenile and adolescent learning that allow the accumulation of knowledge that is such a remarkable feature of behaviorally modern humans, and that is such an important factor in the economic success and complex social structures and relationships of *Homo sapiens* [145]. It may also have encouraged cooperation among individuals to promote the success of their children, grandchildren, and others in their extended communities [128, 131], enabling the technological innovativeness, behavioral flexibility, and exploratory disposition needed to allow *Homo* sapiens to spread throughout the world more successfully than other human lineages [1–3]. Further work is needed to understand the specific functions of the lncRNAs associated with self-awareness that underlie the capacity of modern humans for healthy longevity, prosociality, and creativity. Fuller understanding is greatly needed because of the frequent failure of these beneficial capacities of modern humans to be self-actualized during the Anthropocene [52].

## Supplementary information

Supplementary Methods and Information

Supplementary Figure S1

Supplementary Figure S2

Supplementary Figuee S3

Supplementary Figure S4

Supplementary Figure S5

Supplementary Figure S6

Supplementary Table S1

Supplementary Table S2

Supplementary Table S3

Supplementary Table S4

Supplementary Table S5

Supplementary Table S6

Supplementary Table S7

Supplementary Tables S8 - S12

Supplementary Table S13

Supplementary Table S14

## Data Availability

The Young Finns Study granted data access to CRC and IZ by a Materials Transfer Agreement.
