# Supplementary material for: Evolution of genetic networks for human creativity
Source: Mol Psychiatry. 2021 Apr 21;27(1):354–76. doi: 10.1038/s41380-021-01097-y (PMC8960414; doi:10.1038/s41380-021-01097-y)
Supplement: Supplementary file 1 — Supplementary Methods and Information [file 41380_2021_1097_MOESM1_ESM.pdf]

Supplementary Information for

Title: Evolution of Genetic Networks for Human Creativity

Authors: I. Zwir[1,2#], C. Del-Val[2#], M. Hintsanen[3], K.M. Cloninger[4], R. Romero-Zaliz[2], A. Mesa[2], J. Arnedo[2], R. Salas[5], G.F. Poblete[5,6], E. Raitoharju[7], O. Raitakari[8], L. Keltikangas-Järvinen[9], G. de Erausquin[10], I. Tattersall[11], T. Lehtimäki[7], C. R. Cloninger[1,4*]

# these authors contributed equally

* corresponding author

Running Title: Evolution of Human Creativity

Affiliations:

[1] Washington University School of Medicine, Department of Psychiatry, St. Louis, Missouri

[2] University of Granada, Department of Computer Science, Granada, Spain

[3] University of Oulu, Unit of Psychology, Faculty of Education, Oulu, Finland

[4] Anthropedia Foundation, St. Louis, Missouri

[5] The Menninger Clinic and Baylor College of Medicine, Houston, Texas, USA

[6] University of Buenos Aires, Faculty of Exact Science, Buenos Aires, Argentina

[7] Department of Clinical Chemistry, Fimlab Laboratories, and Finnish Cardiovascular Research Center - Tampere, Faculty of Medicine and Health Technology, Tampere University, Tampere, Finland

[8] Center for Population Health Research, University of Turku and Turku

University Hospital; Research Center of Applied and Preventive Cardiovascular Medicine, University of Turku; Department of Clinical Physiology and Nuclear Medicine, Turku University Hospital, Turku, Finland

[9] University of Helsinki, Department of Psychology and Logopedics, Helsinki, Finland

[10] University of Texas San Antonio, Long School of Medicine, Department of Psychiatry, The Glenn Briggs Institute of Alzheimer's and Neurodegenerative Disorders, San Antonio, Texas

[11] American Museum of Natural History, New York, New York

Corresponding Author: C. Robert Cloninger, MD, PhD, Washington University School of Medicine, Department of Psychiatry - Campus Box 8134, 660 South Euclid Avenue, St. Louis, MO 63110; 314-374-7187 (phone), crcloninger44@gmail.com (email)

This PDF file includes:

Supplementary Information Outline

Assessment of Human Personality at Three levels of Organization..............................3
Dimensions and Facets of Temperament and Character....................................3
Multi-trait Temperament or Character Profiles & Indices of Well-being...........4
Joint Temperament-Character networks..5
Gene Identification and Annotation in Modern Humans..6
Genome Coverage..7
Comparative Genomic Analysis...8
Description of Genomic Samples...8
Neanderthal Genome Project Analysis...10
High-coverage Altai Neanderthal Genome Analysis...11
Vindija 33.19 and Chagyrskaya Neanderthal genome analyses..............13

General Statistical Methods..15
One-Way Analysis of Variance for Independent and Correlated Samples.........15
Estimation of ANOVA Effect Size...15
The One-Way ANOVA for Independent Samples....................................17
The One-Way ANOVA for Correlated Samples..17
The Tukey HSD Test...18
Genotypic Estimation of Behavioral Modernity of Neanderthals.......................19
Horizontal Gene Transfer Analysis...20
Gene Expression Analysis..21
Derived Allele Frequency (DAF score) Analysis..21
Analysis of selection on personality-related lincRNAs.......................................22
Analysis of expression of personality-related genes in human brain regions....23

Figs. S1 to S6
Tables S1 to S14

Assessment of Human Personality at three levels of organization

Dimensions and Facets of Temperament and Character

The facets (subscales) of the TCI dimensions are summarized in Table S1 along with descriptors of high and low scorers; research documenting the validity of these descriptions and their neurobiological basis are detailed elsewhere[1-4]. Harm Avoidance, an indicator of passive avoidance conditioning, is the sum of its four subscales in which high scores indicate pessimism and worry, fear of uncertainty, shyness, and rapid fatigability. Low harm avoidance reduces sensitivity to harsh conditions, but recklessness can be lethal. Persistence, an indicator of the partial reinforcement extinction effect in which intermittently rewarded behaviors are more slowly extinguished than continuously reinforced behavior, is measured by its four subscales describing determination to succeed despite frustration and fatigue. High Persistence can be beneficial when reward conditions are stable, but it can be counterproductive

without Self-directedness and insight into signs of changing future conditions. Self-Transcendence is an indicator of identification with other people, nature, or the universe as a whole, so Self-transcendent individuals exhibit prosocial behaviors, such as being trusting, altruistic, and willing to make sacrifices that are not beneficial to themselves individually. In contrast, individuals who are high in Cooperativeness,, but not Self-transcendence, may be helpful and empathic at times, but only when it is also to their own benefit.

Table S1 about here

Multi-trait temperament and character profiles & Indices of Well-being

Our prior analyses of character and of temperament showed that temperament and character are each complex in the sense that different genetic and environmental processes can result in the same personality outcome. We found that the genetic antecedents of personality code for predisposition to specific multi-trait profiles of temperament and of character, as detailed below and elsewhere[3-7]. For example, high scores on all three TCI character traits identifies one of the character clusters called the "creative character profile" of high Self-directedness, Cooperativeness, and Self-transcendence), as validated by tests of creativity, such as tests of divergent thinking[8,9] and other clinical and developmental research[10-13].

The product of all three TCI character scales (SD x CO x ST), which is high in individuals with the creative character profile, also provides a useful indicator of well-

being, including its physical, emotional, cognitive, social, and spiritual aspects. It has been validated in multiple cultures, including our Finnish sample [13,14]. In the Finnish sample we confirmed its validity as an indicator of well-being with independent measures of positive affect balance, social support, physical behaviors (exercise, smoking, diet) and objective laboratory findings for ideal health recommended by the American Heart Association, as summarized elsewhere[14] and in Supplementary Table S2. Although it is also an effective indicator of verbal and figural creativity, it neglects some of the other motivational components of creative achievement measured by multi-trait temperament profiles, particularly the reliable profile, which is specified by low Novelty Seeking, high Reward Dependence, and high Persistence), as discussed in detail elsewhere[15,16].

see Supplementary Table S2

Joint Temperament-Character Networks

In prior work we also found that the multi-trait temperament profiles and multi-trait character profiles are functionally integrated into joint networks through gene-environment interactions over the course of lifespan development[14,17]. In other words, character profiles provide the rational insight that guides self-regulation to bring a person's habits in accord with the goals and values. We found that there were 3 nearly disjoint clusters of temperament-character networks that correspond to the three major systems of human learning and memory, as described elsewhere and in the

introduction[14]. The creative-reliable network is composed primarily of individuals with reliable temperaments and creative character profiles, which combines the configuration of features characteristic of individuals with high creative achievement, particularly people with both creative character profiles plus Persistence as empirically shown elsewhere[8,9].

Gene identification and annotation in modern humans

Our sample of modern human subjects (Sapiens) was the Young Finns Study, an epidemiological study of 2,149 healthy Finnish subjects[18]. All subjects had thorough standardized genotypic, environmental, and phenotypic assessments, including administration of the Temperament and Character Inventory (TCI)[3,4]. Information about the genotyping and identification of Single Nucleotide Polymorphisms (SNPs) associated with human personality is detailed elsewhere[3,4]. Briefly, Phenotype-Genotype Many-to-many Relations Analysis (PGMRA) was used in GWAS to account for the natural clustering of individuals with particular configuration of SNPs in SNP sets [19]. The PGMRA accounts for Linkage Disequilibrium efficiently (i.e., without loss of information about complex genotypic-phenotypic relations)[20]. Statistical analysis correcting for multiple comparisons, and gender and ethnicity as covariates of the SNP sets was performed by SNP Kernel Association Test (SKAT) to evaluate significance of association using standard thresholds for genome-wide association studies[21,22].

The study has been carried out with 972 genes mapped to SNP sets of the three phenotypic networks: Creative-Reliable, Organized-Reliable, and Emotional-Unregulated (Supplementary Figure S1)[14]. We refer to the corresponding genotypic networks as the Self-awareness, Self-control, and Emotional Reactivity networks, respectively (Supplementary Table S1 and Figures S2). The genotypic networks are strong indicators of the corresponding phenotypic networks (Supplementary Figure S3)[14]. The annotations of individual genes were obtained using the perl API of Ensembl [23] versions 87-92 (see Table S3) and classified according to their biotype distinguishing between protein coding genes, non-coding RNA genes and pseudogenes (see Table S4).

See Tables S2, S3, and Figures S1, S2, S3

Genome coverage

The coverage (or depth) in DNA sequencing is defined as the number of unique reads that include a given nucleotide in the reconstructed sequence. Deep sequencing refers to the general concept of aiming for a high number of unique reads of each region of a sequence.

The average coverage for a whole genome can be calculated from the length of the original genome (G), the number of reads (N), and the average read length (L) as $(N \times L)/G$. For example, a hypothetical genome with 2,000 base pairs reconstructed from 8 reads with an average length of 500 nucleotides will have 2-fold redundancy. This parameter also enables one to estimate other quantities, such as the percentage of the

genome covered by reads (sometimes called coverage). A high coverage in shotgun sequencing is desired because it can overcome errors in base calling and assembly.

Comparative Genomic Analysis

Description of the genomic samples

Chimpanzee orthologs for the 972 genes accounting for personality in modern *Homo sapiens* were obtained by accessing the CHIMP2.1.4 database, which uses the *Pan troglodytes* model (7/20/16) built from genome (v.2.1.4) with gene model files (R.89) from Ensembl using the Perl API [24]. The orthologous genes for the rest of primates (Bonobo, Chimpanzee, Gibbon, Gorilla, Human, Macaque, Marmoset, and Orangutan,) were obtained using programmatic access to resources in Ensembl [25].

In contrast to the Chimpanzee genome, there is not an available catalog of genes derived from the Neanderthal genome[26,27]. The only standard annotated resource available is from the Neanderthal Genome Project, which is based on six samples from members of *Homo neanderthalensis* [26]. 98% of the genome sequence in this resource comes from three specimens found in the Vindija Cave in Croatia, and known as Vi33.16, Vi33.25 and Vi33.26. However, these are low quality (~1.2-fold total coverage) sequences [26,28]. The other 2% of the genome is derived from three other Neanderthal sequences. One of these, the Mez1 was from Mezmaiskaya Cave in the Altai Mountains, Russia, which is a low-quality genome sequence (~0.5-fold genomic coverage) [27]. 0.1% of

the genome was taken from the specimen called Feld1, which was found in the Neander Valley in Germany, and another 0.1% was recovered from a specimen of the Sid1253 individual from Sidron Cave in Asturias, Spain [29]. In addition, chromosome 21 and exome sequences have been generated from another individual from Vindija Cave (Vi33.15) and another from Sidron Cave in Spain. [A high coverage genome from the Vindija cave (Vi33.19) has also been produced and corresponds to the same individual as 33.15[30].]

The best-quality data about Neanderthal genome is the complete genome sequence for the "Altai Neanderthal" from the Denisova Cave in the Altai Mountains in Russia (coverage ~50x)[27]. The two other high-coverage Neanderthal genomes currently available are from the Vindija 33.19 sample (coverage ~30x)[30] and from the distal manual phalanx of an individual found in the Chagyrskaya cave in Russia (~28x)[31]. Although there is some high-coverage genomic data about Denisovans, we chose to focus only on Neanderthals because Denisovans form a single clade with Neanderthals and more information is available about the genomes and behavior of Neanderthals. There is no genome annotation available for these high-coverage individuals, but alignment and raw data are freely available under the Ft. Lauderdale principles (http://cdna.eva.mpg.de/neandertal/).

The diversity and quality of Neanderthal genome sequences, especially from the center of their geographical range and from the time close to when they were estimated

to have mixed with modern humans, may limit our ability to reconstruct their history and the extent of their genetic contribution to present day humans[30] (Figure S6). Neanderthals lived in Vindija Cave in Croatia until relatively late in their history [26,28]. The cave has yielded Neanderthal and animal bones, many of them too fragmentary to determine from their morphology from what species they are derived. Notably, DNA preservation in Vindija Cave is relatively good and allowed the determination of Pleistocene nuclear DNA from a cave bear, a Neanderthal genome, and exome and chromosome 21 sequences [26-30].

We estimated the genes associated with personality in modern Eurasian *Homo sapiens* that were also present in the Neanderthal, including separate analyses of the data of the Neanderthal Genome Project[26], the Altai Neanderthal[27], the Vindija 33.19[30] and the Chagyrskaya genome[31] (http://cdna.eva.mpg.de/neandertal/Chagyrskaya). The estimated ages of the specimens are 50 - 65 kya for Vindija Neanderthal, 80 kya for the Chargyskaya Neanderthal, and 120-130 kya for Altai Neanderthal[27,30,31].

Neanderthal Genome Project analysis

The comparison of the aligned sequences corresponding to the Neanderthal genome with those sequences derived from the human genome allowed the identification of SNPs using the UCSC Genome Browser (http://genome.ucsc.edu).

First, to estimate the genes associated with personality in modern *Homo sapiens* that were also present in the Neanderthal genome, we collected the full sets of Single

Nucleotide Polymorphism changes and insertion/deletions [27]. We assumed that all genes that appear in the dataset of Prüfer and colleagues [27] are present in both Neanderthal and Sapiens genomes. The data are available at the Max Planck Gesellschaft (http://cdna.eva.mpg.de/Neanderthal/altai/) and at the FTP server at the EBI (ftp://ftp.ebi.ac.uk/pub/databases/ensembl/Neanderthal).

Second, we manually analyzed the resulting set of missing genes using the UCSC browser with all six available Neanderthal sequences from the Neanderthal Genome Project. We considered that a modern *Homo sapiens* gene was present in the Neanderthal genome if three out of the six Neanderthal sequences were aligned and overlapped the genomic region of that gene in modern *Homo sapiens*.

High Coverage Altai Neanderthal genome analysis

The comparison of the aligned sequences corresponding to the high coverage Altai Neanderthal genome with those sequences derived from the Sapiens genome allowed Prüfer et al (2014) to identify derived changes in the human lineage available as a set of single nucleotide changes (SNCs) and as a set of insertion/deletions (InDel). We have used the EnsemblGenes subset of the catalogs which correspond to changes in Ensembl genes.

First, to estimate the genes associated with personality in modern *Homo sapiens* that were also present in the high coverage Altai Neanderthal genome, we used the full sets in the human catalog of SNCs and insertion/deletions provided by Prüfer et al

(2014)
(http://cdna.eva.mpg.de/neandertal/altai/AltaiNeandertal/catalog/HumanSpecific/). We assumed that all personality-related genes that appear with a SNC or with an InDel in these datasets are present in both genomes independently of the consequences that the SNC/InDel could produce in the gene.

We generated four column data for the human personality genes: 1) *Altai Prüfer Ensembl catalog* that uses only the SNC that occurs within a gene including 5′, 3′ UTRs, splicing sites and codons; 2) *Altai derived InDels* that uses the full set of InDels in the human catalog 3) *Altai derived SNC* uses the complete SNC catalogs taking into account SNCs that are located in intergenic regions close to a certain gene 4) A summary column with an entry of "Yes" if any of the previously described columns contain a gene found in the human personality-related gene set.

Results show full agreement in the number of human genes found (n = 267) in the Neanderthal genome project[26] and in the high coverage Altai Neanderthal genome[27]. Prüfer and colleagues ranked all single-nucleotide changes and all small InDels (<12 bp) using the "Combined Annotation Dependent Depletion" score (CADD), (http://krishna.gs.washington.edu/download/CADD/v0.5/) as PHRED-scaled scores, also called C-scores. This PHRED scalation is based on the rank that the variant occupies relative to all possible substitution changes in the genome. A change with a PHRED-scaled score of 20 or greater indicates a high C-score that could be interpreted

as “disruptive”, which provides a way to prioritize them for future experimental studies to evaluate their effects and possible functional changes in these genes between modern Humans and Neanderthals. We examined the 30 genes in the aligned regions between modern humans and Altai Neanderthal with the highest C-score described by Prüfer and colleagues[27]. Among them we find only one gene in the human personality-related gene set, ENSG00000166501 (PRKCB), a protein kinase of the PKC family that has been suggested to regulate neuronal functions and correlate fear-induced conflict behavior after stress in mice. The high C-score may indicate a disruptive change likely to change the function of the gene in the Altai Neanderthal from its function in modern humans, so the C-scores also confirm our initial findings with the 2010 draft Neanderthal genome regarding our 972 genes of interest.

Vindija Neanderthal sample 33.19 and Chagyrskaya Neanderthal genome analyses

We selected these two genomes because of the high coverage of their data. The the Vindija Neanderthal sample 33.19 (Vindija33.19) from the Vindija Cave in Croatia with a genome coverage of 30x[30] and the Neanderthal genome from the Chagyrskaya Cave in the Altai Mountains, Russia (Chargyskaya)[31] with a coverage of 28x, which belongs to the distal manual phalanx of a female. First, we collected the filtered Bed files containing the general filters advised to be applied to any study with the Vindija 33.19 genome (http://cdna.eva.mpg.de/neandertal/Vindija/FilterBed/) and the Chagyrskaya

genome (http://cdna.eva.mpg.de/neandertal/Chagyrskaya/FilterBed). Then we generated chromosome-based bed files of the genes associated with personality mapped to the Genome Reference Consortium Human Build 37 (GRCh37) hg19 assembly. Bedtools2.26.0 [32] was then used to calculate the intersection between the human personality genes bed files and the filtered bed files of Vindija33.19's and Chagyrskaya's genomes independently. Results were analyzed to calculate the percentage of each personality human genes covered in each of the selected Neantherthal genomes using our own scripts to calculate the base coverage of each human gene in the corresponding filtered hg19 alignments.

Our policy consisted of comparing which of the 972 genes accounting for personality in modern humans we could also identify in the draft Neanderthal genome (Consortium 2010) or any of 3 Neanderthal genomes that have been subsequently characterized with high-coverage: the high-coverage Vindija 33.19, the Altai Neanderthal (Prufer 2014) and the Chargyrskaya Neanderthal (Table S5). This provided an unbiased approach that considered all available information. We observed differences in the percentage of coverage found between high-coverage genomes in some genes (see Table S5), which can be due to differences between genomes in their alignment regions that do not pass the recommended filters. For the 972 genes associated with personality in modern humans, 267 genes were not found in any

Neanderthal genome, including the 3 high-coverage genomes. This finding is therefore robustly replicable, but absence of proof is never proof of absence, so we tested the functional significance of the genes found only in modern humans by examining their effects on selection and on gene-expression (see below).

see Supplementary Table S5

[Note: numbering of Supplementary Tables and Figures follows the order in which they are referred to in the main text]

Some illustrative examples are provided in Supplementary Figure S6 of genes missing in Neanderthals but present in Chimpanzees and Sapiens (MIR6760, Figure S6A, and MIR6761, Figure S6B) and of a gene present in Sapiens but missing in chimpanzees and Neanderthals (BIRC8, Figure S6C). BIRC8 protects against programmed cell death under conditions of oxidative stress as a unique component of the self-awareness network in modern humans (Supplementary Table S3).

see main article for discussion of Supplementary Figures S4, S5

see Supplementary Figure S6A,B,C

General Statistical Methods

One-Way Analysis of Variance for Independent and Correlated Samples

We used the analysis of variance to test the null hypothesis that the three studied networks are similar in terms of the genes that compose them within one species and across species (Sapiens, Chimpanzee, and Neanderthal). To do so, we utilized both the

ANOVA for independent and correlated ($k = 3$) samples, one per network (Self-awareness vs Self-control vs Emotional Reactivity) in each of the species. Then, we applied the Tukey HSD Test to evaluate the specific differences between pairs of networks (e.g., Creative vs Organized). We established an empirical association between the type of test and the eventual conservation of the genes. For example, correlated samples are implied by vertical inheritance of ancestral genes or continuity in evolution, whereas independent samples suggest that they are randomly drawn (e.g., horizontally acquired genes). We used the ANOVA test as implemented in Concepts & Applications of Inferential Statistics, Richard Lowry 1998-2021, http://vassarstats.net/anova1u.html, rstatix package in R, and Matlab R2017b, Statistical toolbox [33-35].

The ANOVA effect size was calculated as the f value defined by Cohen[36], where he proposed the following interpretation of this value: $f = 0.1$ is a small effect, $f = 0.25$ is a medium effect, and $f = 0.4$ is a large effect (effect size package in R and/or https://webpower.psychstat.org/models/means03/effectsize.php (REF)). See that f can be easily transformed into *eta squared* $= f^2 / (1+f^2)$. All other parameters used in each measurement of ANOVA were calculated as usual[36,37], and specified in Supplementary Tables S8-S12.

See Supplementary Tables S8-S12

The One-Way ANOVA for Independent Samples

This version of ANOVA applies to the case where there is one independent variable and three or more independent samples of subjects, with each sample measured at a different level of the variable. This particular version of the analysis of variance makes the following assumptions about the data that are being fed into it:

1. that the scale on which the dependent variable is measured has the properties of an equal interval scale;
2. that the **k** samples are independently and randomly drawn from the source population(s);
3. that the source population(s) can be reasonably supposed to have a normal distribution; and
4. that the **k** samples have approximately equal variances.

When the samples are of the same size, the analysis of variance is also robust with respect to the assumption that the source populations are normally distributed.

One-Way Analysis of Variance for Correlated Samples

This version of the analysis of variance is an extension of the correlated-samples **t**-test. It has the same structure with the correlated-samples ANOVA, where we have a certain number of subjects, each measured under three or more conditions: A|B|C, A|B|C|D, and so forth. When the analysis involves each subject being measured under each of the k conditions, it is sometimes described as a repeated measures design or a

within subjects design. The utility of the correlated-samples ANOVA is that it is highly effective in removing the extraneous variability that derives from pre-existing individual differences. In some cases individual differences might be the very essence of the phenomena that are of interest, but there are also many situations where they are merely irrelevant clutter.

The Tukey HSD Test

Tukey's range HSD (Honestly Significant Difference) test is also called the Tukey–Kramer method. It is a single-step multiple-comparison procedure and statistical test. We use it here in conjunction with an ANOVA (post-hoc analysis) to find means that are significantly different from each other. It compares all possible pairs of means, and it is based on a studentized range distribution (q) (this distribution is similar to the distribution of t from the t-test. See below). The Tukey HSD tests should not be confused with the Tukey Mean Difference tests, which is also known as the Bland–Altman diagram. Tukey's test compares the means of every treatment to the means of every other treatment; that is, it applies simultaneously to the set of all pairwise comparisons and identifies any difference between two means that is greater than the expected standard error. In other words, the Tukey method is conservative when there are unequal sample sizes. We used the Tukey's test as implemented in Concepts & Applications of Inferential Statistics, Richard Lowry 1998-2021, http://vassarstats.net/anova1u.html and rstatix package in R.

Genotypic Estimation of the Behavioral Modernity of Neanderthals

The number of individual genes that Neanderthals shared with modern humans may not be an adequate indicator of their impact on creativity and other aspects of modern human functioning. Therefore, we evaluated the impact of genes on the predisposition to modern human well-being as an indicator of behavioral modernity by estimating their relative roles in specific SNP sets to take into account the interactions among coordinated sets of genes that impact well-being.

In order to extract prototypical samples of humans with distinctive Neanderthal-like features and distinctive Sapiens-like features, we first identified the 267 genes found only in Sapiens and the 148 genes Neanderthals shared with Sapiens, excluding genes present in chimpanzees, which are listed in Supplementary Table S3. Then we cross-correlated these genes with the original SNP- sets in which they had been detected in relation to character and/or temperament, which are listed and described in Supplementary Tables S6 of our prior reports about character[3] and temperament[4]. SNP-sets are clusters specified by individual humans who have particular groups of SNPs. We selected SNP sets found in the genotypic networks for self-awareness, self-control, and emotional reactivity, for which we already had measured the associated levels of functioning in modern humans, including two indices (well-being and resilience from ill-being) as provided in Supplementary Tables S7 of our description of these joint

character-temperament networks[14]. Then from the measures of well-being that we had for SNP sets that contained one or more of the 148 genes that Neanderthals shared with Sapiens but not chimpanzees, we estimated the mean well-being of Neanderthal-like humans by weighting the well-being of people in those individual SNP-sets by the proportion of genes present in Neanderthals compared to Sapiens in that SNP-set. Likewise, we estimated the mean well-being of prototypical Sapiens-like humans from the measures of well-being of people in SNP-sets that contained one or more of the 267 genes found only in modern humans. Finally, we compared the means levels of well-being in Neanderthal-like humans to Sapiens-like humans using ANOVA statistics, including effect sizes and the probability of differences between the means (Supplementary Table S10).

Finally, we estimated the relative genotypic modernity of these prototypes for the two species from the ratio of their mean levels of well-being. For example, in Table S10, for genes in SNP-sets in the self-awareness genotypic networks, the mean well-being of Neanderthal-like individuals (viz 5.35) was 70% of that for Sapiens-like individuals (viz 7.6).

Horizontal Gene Transfer analysis

In order to determine if genes mapped to the three phenotypic networks could have been horizontally acquired, we calculated their overlap to the regions of horizontal gene transfer (HGT) identified by Huang and colleagues [38] in the human reference

genome hg 19 [39]. Huang and colleagues specified different regions depending on the similarity threshold between vertebrates and the human genome reference hg19 (40%, 50 %, 60%) and the length of the corresponding coverage (40%, 20%, 0). We used those regions with similarity thresholds of 40% and 50%, and length of coverage of 40%, to extract a subset of our 972 genes that match with those specified as likely to result from HGT.

Huang and colleagues identified 642 genes in HGT regions from among 57,905 Ensembl genes. We found 39 of the 972 genes related to personality were located in the HGT regions identified by Huang and colleagues. These are described in Table S3 according to their known functions, biotype, association with the three genotypic networks, and presence in Neanderthals and/or Sapiens.

See Supplementary Table S3

Gene expression analysis

Gene expression analyses were carried out using ArrayExpress [40] through programmatically provided access.

Derived Allele Frequency (DAF score)

The Derived Allele Frequency (DAF) score [41] was calculated for all the lincRNA genes present uniquely in modern humans and thus lacking an ortholog. To calculate the DAF scores for all lincRNAs, including their exons and promoters, we used the AnnLoc tool (http://annolnc.cbi.pku.edu.cn) that enables the systematic annotation of

these genes based on certain evolutionary parameters. A DAF score lower than 0.1 indicates purifying (negative) selection for humans.

Analysis of selection on personality-related lincRNAs

We found 127 lincRNAs without orthologs associated with personality, including 68 present only in modern humans (n = 68) (Supplementary Tables S3 and S5; also see Figure S4) and 59 present in Neanderthals (n = 59) (Supplementary Table S4). Information about the DAF scores was available for 60 lincRNAs unique to modern humans (Supplementary Table S6) and 53 present in Neanderthals (Supplementary Table S7),

see Supplementary Tables S3 to S7

[note: numbering order follows references to Supplementary Tables and Figures in the main text)]

Among the 60 lincRNAs unique to modern humans, those with DAF > 0.1 are more frequent than others for both their promoters (40 of 60 vs 12 of 60, ANOVA, $F(1,110) = 30.23$, $p < 0.0001$) and their exons (34 of 60 vs 18 of 60, ANOVA, $F(1,110) = 9.78$, $p < 0.0022$, Supplementary Tables S6 and S12). Their promoters had DAF > 0.1 slightly more often than their exons (67% vs 56%, ANOVA, $F(1,102) = 4.54$, $p < 0.03$), suggesting that positive selection is acting on regulatory functions in modern humans.

Among the 53 personality-related lincRNAs without orthologs that were present in Neanderthals for which a DAF could be calculated (Supplementary Table S7), we

found that 76% had promoters under positive selection (32 with DAF > 0.1 vs 10 others, ANOVA, $F (1,92) = 45.35$, $p < 0.0001$). Likewise 64% had exons under positive selection (27 with DAF > 0.1 vs 15 others, $F (1,94) = 11.75$, $p < 0.0019$). In Neanderthals, the proportion of promoters with DAF > 0.1 did not differ significantly from the proportion of exons (76% - 64%, not significant).

We also compared the averages of DAF scores above and below the 0.1 threshold in modern humans and Neanderthals in order to further characterize the patterns of selection by considering the irregular shape of the distribution of DAF scores. The difference between the average of the DAF scores of all promoters was 21% higher than the average of DAF scores of all exons of lincRNAs in modern humans, whereas it was 9% higher in Neanderthals. For lincRNAs with DAF > 0.1, the average of all DAF scores was slightly greater in promoters than exons in modern humans (0.27 vs 0.23), but did not differ in Neanderthals (0.26 vs 0.27). For lincRNAs with lower DAF (= or < 0.1), there was no significant difference between the average DAF score in promoters vs exons in either modern humans (0.05 vs 0.04) or Neanderthals (0.07 vs 0.06).

Analysis of expression of personality-related genes in Human Brain regions

Lists of genes that mapped to Character-related SNP sets or to Temperament-related SNP sets primarily in the self-awareness network (i.e., G_12_1, G_20_2, G_28_10, G_33_33, G_42_39, G_20_3, G_19_5, G_28_11, G_3_2, G_33_15, G_9_8, G_28_15) and secondarily in the self-control network (i.e., G_12_8, G_13_10, G_21_18,

G_8_8, G_3_1) were analyzed using Process Genes List (PGL) [42]. All genes were considered if they were present in a SNP set that was significantly associated with human personality and contained at least one gene that we found only in modern humans, which meant that they were primarily from the self-awareness network in which most genes found only in Sapiens occurred (Supplementary Table S13). This machine learning method uses the Allen Human Brain Atlas (AHBA) to calculate a normalized average mRNA expression level in each brain region for a specified gene list. Brain regions in which those genes are most significantly co-expressed are named regions of interest (ROIs) (Supplementary Table S14). The regions are named according to the AHBA nomenclature. After ranking these regions, only the first 10 ROIs are considered to make the brain image plot (see Figure 3 in main article), but all regions in which there is significant expression are tabulated (Supplementary Table S14).

Supplementary Tables S13 and S14

Each Allen region is mapped to the automated anatomical labeling (AAL) equivalent region, according to its Montreal Neurological Institute (MNI) coordinates. One Allen region can correspond to into one or more AAL regions. In the same way, one or more Allen regions can be represented by just one AAL region, so the number of regions can vary from the original Allen regions list. If an Allen region is not available in the AAL region, it is ignored and excluded from the procedure. A color scale is assigned to the new list of AAL regions, taking into the account the same rescaled

importance order of the Allen region original list. To indicate the importance on the region the color scale varies from light yellow to red, where red indicates the highest density of gene co-expression per region.

The analysis has been performed using the default parameters specified in PGL [42]. Given a set of candidate genes, the method first recovers the expression of these genes in the AHBA. Then, PGL uses the Wilcoxon test to determine which regions of the brain have differential gene expression of all or maximal subsets of the genes of interest. Tests that pass with a corrected p-value of 0.05 remain and then are sorted by p-value in ascending order. PGL creates three additional machine learning models to classify co-expressed genes in certain brain regions using Random Forest (RF), Support Vector Machine (SVM), and k-nearest neighbor (KNN) predictive methods that were assembled in PGL as a multi-classifier, which we briefly describe in the following paragraph.

The importance of brain regions (characteristics) in RF was calculated as the decrease in impurity of the node weighted by the probability of reaching that node. The node probability can be calculated by the number of samples reaching the node, divided by the total number of samples. The higher the value, the more important the characteristic. The brain regions were classified accordingly. The other two methods used the step-backward approach to select the most important brain regions and ranked them based on their performance. Finally, the four rankings for a given brain region

were assembled using the average of each performance and re-ranked. We summarize all rankings with above-average levels of expression (final rank scores greater than 0) (Table S14), but for display purposes show only the ten most highly ranked regions (Figure 3 in main text).

We found that the genes that cluster together with the genes found only in modern humans are most densely co-expressed in brain regions that comprise the self-awareness learning network, which provides evidence that the group of genes that we found only in modern humans have objective effects that distinguish modern human brain functions from those of chimpanzees and Neanderthals.

References

1 Cloninger, C. R., Svrakic, D. M. & Przybeck, T. R. A psychobiological model of temperament and character. *Archives of General Psychiatry* **50**, 975-990 (1993).

2 Ravaja, N. & Keltikangas-Jarvinen, K. Cloninger's temperament and character dimensions in young adulthood and their relation to characteristics of parental alcohol use and smoking. *J Stud Alcohol* **62**, 98-104 (2001).

3 Zwir, I. *et al.* Uncovering the complex genetics of human character. *Mol Psychiatry* **25**, 2295-2312, doi:10.1038/s41380-018-0263-6 (2020).

4 Zwir, I. *et al.* Uncovering the complex genetics of human temperament. *Mol Psychiatry* **25**, 2275-2294, doi:10.1038/s41380-018-0264-5 (2020).

5 Cloninger, C. R. & Cloniger, K. M. People create health: effective health promotion is a creative process. *International Journal of Person-centered Medicine* **3**, 114-122, doi:10.5750/1jpcm.v3i2.21 (2013).

6 Cloninger, C. R. & Cloninger, K. M. Development of instruments and evaluative procedures on contributors to illness and health. *International Journal of Person-centered Medicine* **1**, 446-455 (2011).

7 Giakoumaki, S. G. *et al.* Psychometric properties of the Greek TCI-R and its clinical correlates: schizotypy and the self-regulation of affective and cognitive functioning. *PeerJ* **4**, e1830, doi:10.7717/peerj.1830 (2016).

8 Chavez-Eakle, R. A., Lara, M. d. C. & Cruz-Fuentes, C. Personality: A possible bridge between creativity and psychopathology? *Creativity Research Journal* **18**, 27-38, doi:10.1207/s15326934crj1801_4 (2006).

9 Takeuchi, H. *et al.* Mean diffusivity of globus pallidus associated with verbal creativity measured by divergent thinking and creativity-related temperaments in young healthy adults. *Hum Brain Mapp* **36**, 1808-1827, doi:10.1002/hbm.22739 (2015).
10 Cloninger, C. R. *Feeling Good: The science of well-being*. (Oxford University Press, 2004).
11 Cloninger, C. R. What makes people healthy, happy, and fulfilled in the face of current world challenges? *Mens Sana Monogr* **11**, 16-24 (2013).
12 Zohar, A. H., Cloninger, C. R. & McCraty, R. Personality and heart rate variability: Exploring pathways from personality to cardiac coherence and health. *Journal of Social Sciences* **1**, 32-39, doi:10.4236/jss.2013.16007 (2013).
13 Cloninger, C. R. & Zohar, A. H. Personality and the perception of health and happiness. *Journal of Affective Disorders* **128**, 24-32, doi:10.1016/j.jad.2010.06.012 (2011).
14 Zwir, I. *et al.* Three genetic-environmental networks for human personality. *Molecular Psychiatry*, doi:10.1038/s41380-019-0579-x (2021).
15 Cloninger, C. R., Cloninger, K. M., Zwir, I. & Keltigangas-Jarvinen, L. The complex genetics and biology of human temperament: A review of traditional concepts in relation to new molecular findings. *Transactional Psychiatry* **9**, 290, doi:10.1038/s41398-019-0621-4 (2019).
16 Moreira, P. A. S., Inman, R. A. & Cloninger, C. R. Personality networks and behavioral problems: Integrating temperament and character using latent profile and latent class analysis. *Child Psychiatry & Human Development* **2020**, 1-13, doi:10.1007/s10578-020-01063-9 (2020).
17 Zohar, A. H., Zwir, I., Wang, J., Cloninger, C. R. & Anokhin, A. P. The development of temperament and character during adolescence: The processes and phases of change. *Dev Psychopathol* **31**, 601-617, doi:10.1017/S0954579418000159 (2019).
18 Raitakari, O. T. *et al.* Cohort profile: the cardiovascular risk in Young Finns Study. *Int J Epidemiol* **37**, 1220-1226, doi:10.1093/ije/dym225 (2008).
19 Arnedo, J. *et al.* PGMRA: a web server for (phenotype x genotype) many-to-many relation analysis in GWAS. *Nucleic Acids Res* **41**, W142-149, doi:10.1093/nar/gkt496 (2013).
20 Zwir, I. *et al.* Uncovering the Complex Genetics of Human Personality: Response from authors on the PGMRA Model. *Molecular Psychiatry*, doi:10.1038/s41380-019-0399-z (2019).
21 Wu, M. C. *et al.* Powerful SNP-set analysis for case-control genome-wide association studies. *Am J Hum Genet* **86**, 929-942, doi:10.1016/j.ajhg.2010.05.002 (2010).
22 Wu, M. C. *et al.* Rare-variant association testing for sequencing data with the sequence kernel association test. *Am J Hum Genet* **89**, 82-93, doi:10.1016/j.ajhg.2011.05.029 (2011).
23 Yates, A. *et al.* Ensembl 2016. *Nucleic Acids Res* **44**, D710-716, doi:10.1093/nar/gkv1157 (2016).
24 Smedley, D. *et al.* The BioMart community portal: an innovative alternative to large, centralized data repositories. *Nucleic Acids Res* **43**, W589-598, doi:10.1093/nar/gkv350 (2015).
25 Aken, B. L. *et al.* Ensembl 2017. *Nucleic Acids Res* **45**, D635-D642, doi:10.1093/nar/gkw1104 (2017).

26 Green, R. E. *et al.* A draft sequence of the Neandertal genome. *Science* **328**, 710-722, doi:10.1126/science.1188021 (2010).
27 Prufer, K. *et al.* The complete genome sequence of a Neanderthal from the Altai Mountains. *Nature* **505**, 43-49, doi:10.1038/nature12886 (2014).
28 Burbano, H. A. *et al.* Analysis of human accelerated DNA regions using archaic hominin genomes. *PLoS One* **7**, e32877, doi:10.1371/journal.pone.0032877 (2012).
29 Castellano, S. *et al.* Patterns of coding variation in the complete exomes of three Neandertals. *Proc Natl Acad Sci U S A* **111**, 6666-6671, doi:10.1073/pnas.1405138111 (2014).
30 Prüfer, K. *et al.* A high-coverage Neandertal genome from Vindija Cave in Croatia. *Science* **358**, 655-658, doi:10.1126/science.aao1887 (2017).
31 Mafessoni, F. *et al.* A high-coverage Neandertal genome from Chagyrskaya Cave. *Proceedings of the National Academy of Sciences USA* **117**, 15132-15136, doi:10.1073/pnas.2004944117 (2020).
32 Quinlan, A. R. & Hall, I. M. BEDTools: a flexible suite of utilities for comparing genomic features. *Bioinformatics (Oxford, England)* **26**, 841-842, doi:10.1093/bioinformatics/btq033 (2010).
33 Milliken, G. A. & Johnson, D. E. *Analysis of Messy Data*. Vol. 1: Designed Experiments (Chapman & Hall/CRC Press, 1992).
34 Neter, J., Kutner, M. H., Nachtsheim, C. J. & Wasserman, W. *Applied Linear Statistical Models*. (Irwin Press, 1996).
35 Hochberg, Y. & Tamhane, A. C. *Multiple Comparison Procedures*. (John Wiley & Sons, 1987).
36 Cohen, J. *Statistical power analysis for the behavioral sciences*. 2nd edn, (Lawrence Erlbaum, 1988).
37 Zhang, Z. & Yuan, K.-H. *Practical statistical power analysis using Webpower and R*. (ISDSA Press, 2018).
38 Huang, W. *et al.* Widespread of horizontal gene transfer in the human genome. *BMC Genomics* **18**, 274, doi:10.1186/s12864-017-3649-y (2017).
39 Lander, E. S. *et al.* Initial sequencing and analysis of the human genome. *Nature* **409**, 860-921, doi:10.1038/35057062 (2001).
40 Kolesnikov, N. *et al.* ArrayExpress update--simplifying data submissions. *Nucleic Acids Res* **43**, D1113-1116, doi:10.1093/nar/gku1057 (2015).
41 1000 Genomes Project, C. *et al.* A map of human genome variation from population-scale sequencing. *Nature* **467**, 1061-1073, doi:10.1038/nature09534 (2010).
42 Poblete, G. F. *et al.* Process Genes List: An approach to link genetics and human brain imaging. *J Neurosci Methods* **339**, doi:10.106/j.jneumeth.2020.108695 (2020).

Supplementary Figure Legends

Fig. S1. The phenotypic architecture of personality: Relationships among Temperament and Character Sets are naturally partitioned into three sub-networks using bidirectional-clustering techniques: Creative-reliable (violet), Organized-reliable (blue) and Emotional-unreliable (orange).

Figure S2. Relationships among SNP sets associated with Temperament and Character Sets composing the three networks shown in Figure 1: Self-awareness (violet), Self-control (blue) and Emotional Reactivity (orange).

Figure S3. Correlation between the phenotypic (Figure S1) and the genotypic (Figure S2) networks (p value < 6E-52, Hypergeometric statistics): Color codes indicate weak (red) to strong (green) statistical significance. The size of the circles indicates the number of coincident phenotypic-genotypic relationships.

Figure S4. (A) Clustering analysis of the genes within the 3 networks that have orthologs in other species. (B) Distribution in the 3 networks of lincRNAs found in modern humans and/or Neanderthals. (C) Distribution in the 3 networks of lincRNAs that are found only in modern humans.

Figure S5. Observed variability in well-being and ill-being indices in each of the three phenotypic networks of modern human beings: Well-being in the Creative-reliable phenotypic network associated with the Self-awareness genotypic network (A), Organized-reliable phenotypic network associated with Self-control genotypic network (B), and Emotional-unreliable phenotypic network associated with Emotional Reactivity genotypic network (C), and Ill-being in the Creative-reliable network (D), Organized-reliable network (E), and Emotional-unreliable network (F).

Figure S6. Examples of genes present in Chimpanzee and modern Humans and missing in Neanderthals (A-B): The (A) MIR6760 and (B) MIR6761 genes as seen by the UCSC browser revealing the six tracks of the Neanderthal sequences (black and grey color), the Chimpanzee sequences are indicated as track Chimp net, the color changes according to the chromosome localization. Human tracks are: RefSeq curated (light blue), Ensembl genes (red), UCSC genes (dark blue). Although some Neanderthal specimen sequences are present, they do not cover more than half of the human query gene (e.g., the low coverage of the Vindija sequences and the few reads available from the other specimens do not allow us to say that those genes are present). (C). The BIRC8 genes as an example of a gene present in Human and missing in Neanderthal and Chimp but with orthologs in other primates. The UCSC browser figure reveals the six tracks of the Neanderthal sequences (black and grey color), the Chimpanzee sequences are indicated as track Chimp net, the color changes according to the chromosome

localization. Human tracks are: RefSeq curated (light blue), Ensembl genes (red), UCSC genes (dark blue). Although some Neanderthal specimen sequences are present, they do not cover more than half of the human query gene (e.g., the low coverage of the Vindija sequences and the few reads available from the other specimens do not allow us to say that those genes are present).

Supplementary Tables

(Legends are all shown here but most tables are large and provided separately)

Table S1. Descriptors for high and low scorers on TCI subscales.

Table S2. Comparison of physical, emotional, social, and cognitive indicators of health of people in 3 personality networks in Young Finns Study (n = 2126).

* Tukey tests: 1, Creative; 2, Organized; 3; Emotional.

Table S3. Description of the 972 genes belonging to the three genotypic networks. *indicates genes mapped by a large SNP set G_3_1 distinguishing healthy vs unhealthy personality, and # indicates genes that are recognized by only one SNP set. Presence of genes in networks replicated in Chimpanzee and Neanderthal are shown. Orthologs, Paralogs and HGT genes are highlighted.

Table S4. Distribution of types of genes found in the 3 genotypic networks of modern humans that are also present in Chimpanzees and Neanderthals.

Table S5. Analysis of 267 genes found in modern humans and not in chimpanzees or Neanderthals according to The Neanderthal Genome Project, Prüfer Analyses, and Chagyrskaya Project. *percentage of bp coverage in the genomes.

Table S6. Counts of lincRNA genes unique to modern Eurasian humans in terms of the DAF scores of their promoters and exons

Table S7. Counts of lincRNA genes present in Neanderthals in terms of the DAF scores of their promoters and exons.

Table S8. Data summary of One-Way ANOVA analysis for the significance of the differences in the number of the 972 genes associated with personality in modern humans among the three species (ANOVA, $p < 0.0001$) with contrasts of numbers of these genes in pairs of the species depending on whether the samples are assumed to be correlated or independent. Significance of each comparison corrected for number of tests is shown (Table 2). The species include modern M1=Homo sapiens ("Sapiens"), M2=Pan troglodytes (Chimpanzees), and M3=Homo neanderthalensis ("Neanderthals"). Tukey HSD Test was used to pairwise comparisons. f is the Cohen effect size (see Supplementary Methods).

Table S9. Data summary of One-Way ANOVA analysis for the significance of the proportions of genes for the three networks between chimpanzees and Neanderthals. Samples were unbiasely treated as independent. Comparisons include all or non-redundant genes. f is the Cohen effect size (see Supplementary Methods).

Table S10. Data summary of One-Way ANOVA analysis for the significance of the well- and ill-being of Neanderthal-like compared to Sapiens-like SNP-sets. f is the Cohen effect size. Samples were considered correlated. (See Supplementary Methods.)

Table S11. Data summary of One-Way ANOVA analysis for the significance of the lncRNAs and pseudogenes distinguishing the types of genes found in the three personality networks. f is the Cohen effect size. Samples were considered independent (See Supplementary Methods).

Table S12. Data summary of One-Way ANOVA analysis for the significance of the DAF values. f is the Cohen effect size. Samples were considered correlated. (See Supplementary Methods.)

Table S13. Selection of groups of genes mapped to personality-related SNP sets that include at least one gene found only in modern humans. Results showing genes in significantly expressed regions of interest and their association with the Self-awareness

and Self-control learning networks. Two large SNP sets, (G_3_1) and (G_8_8) related to organized character in the self-control network were included. (G_8_8) is also related to the reliable temperament.

Table S14. Regions of Interest (ROIs) identified by using the Process Genes List (PGL) program with the Allen Human Brain Atlas. Gene Match indicates only human genes from the personality SNP sets displaying significant differential expression in distinct brain areas. *1 are genes matched with a large SNP set (G_3_1). *2 are genes matched with another large SNP set (G_8_8). "Multiple" indicates Self-awareness and Self-control networks.
