# Supplementary figures and images for: Evolution of genetic networks for human creativity
Source: Mol Psychiatry. 2021 Apr 21;27(1):354–76. doi: 10.1038/s41380-021-01097-y (PMC8960414; doi:10.1038/s41380-021-01097-y)

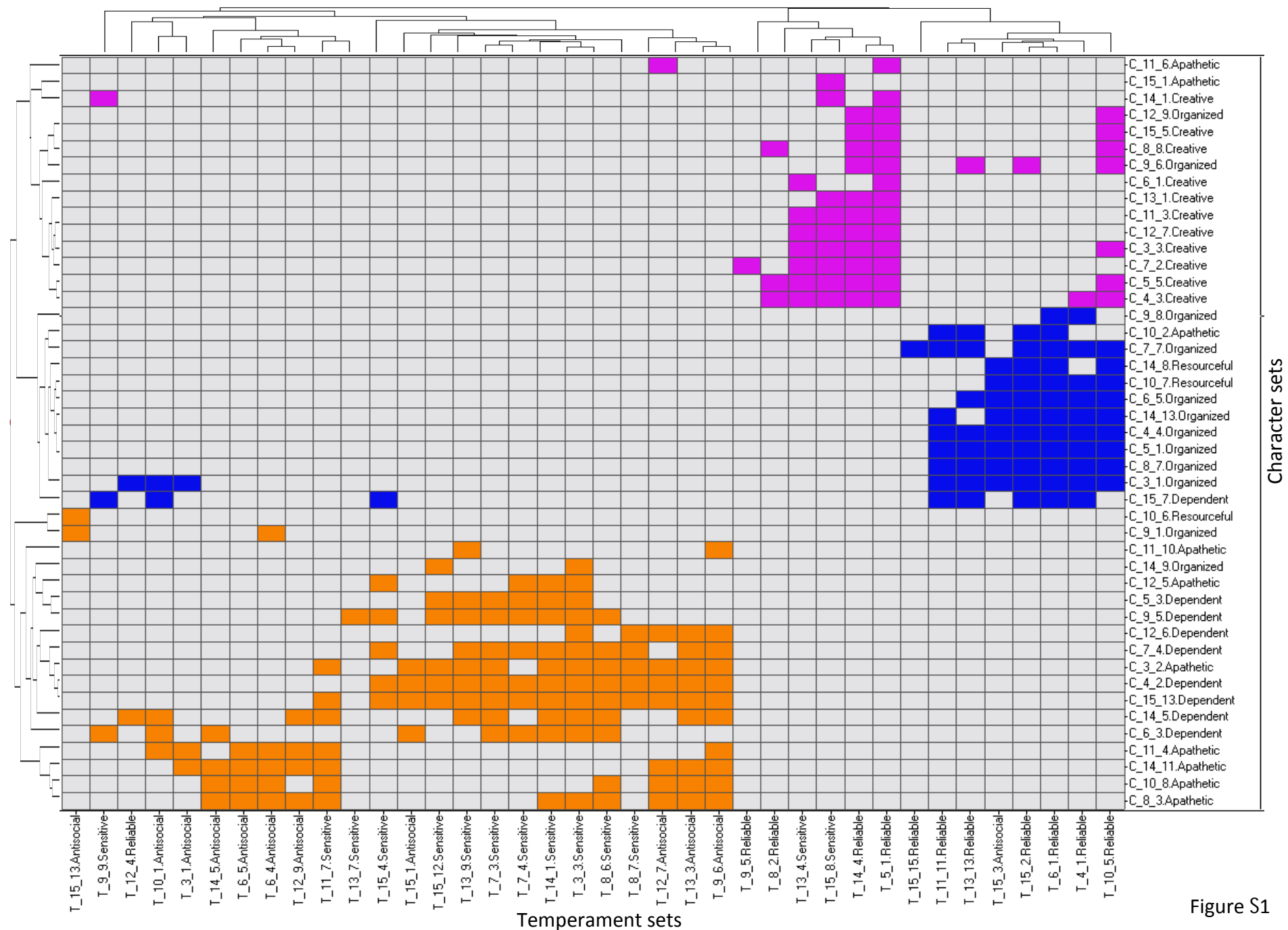

Figure S1

Supplement: Supplementary file 2 — Supplementary Figure S1 [file 41380_2021_1097_MOESM2_ESM.pdf]

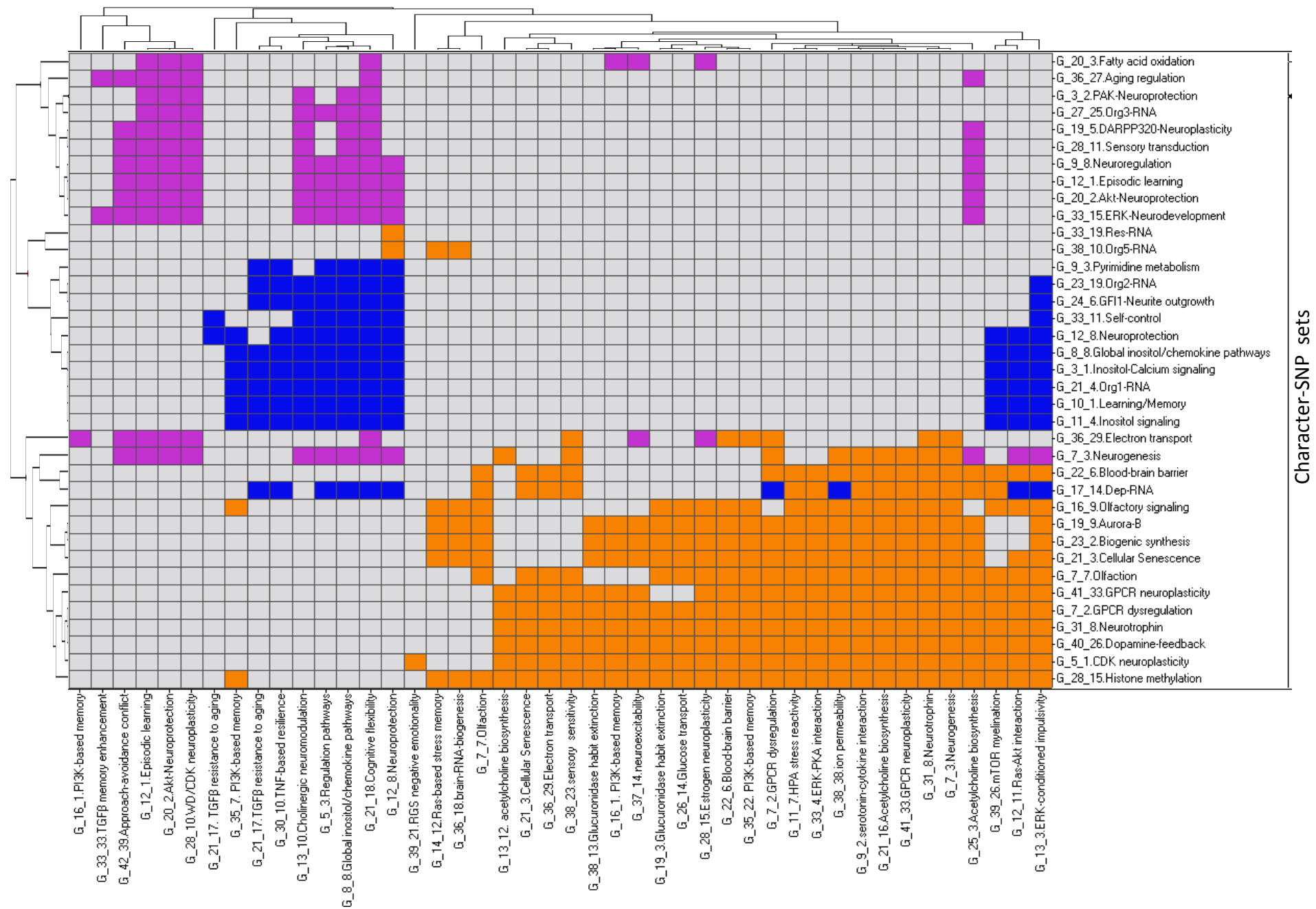

Temperament-SNP sets

Figure S2

Supplement: Supplementary file 3 — Supplementary Figure S2 [file 41380_2021_1097_MOESM3_ESM.pdf]

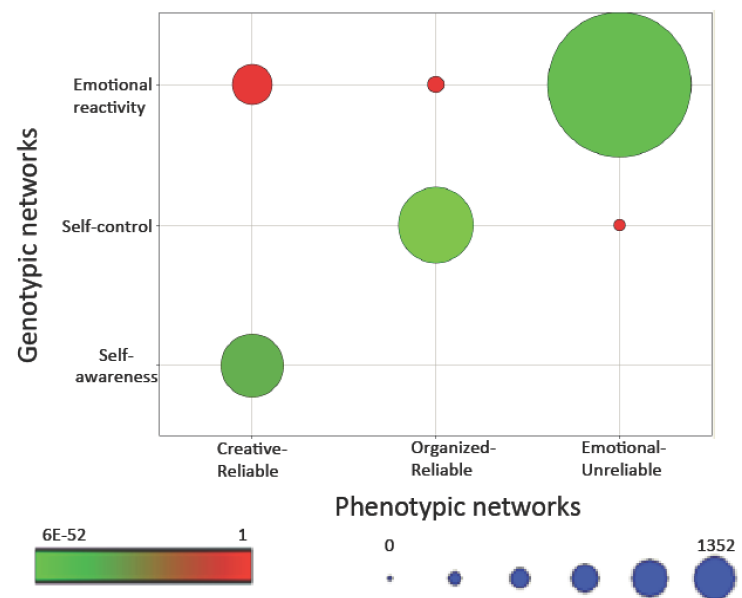

Fig. S3

Supplement: Supplementary file 4 — Supplementary Figuee S3 [file 41380_2021_1097_MOESM4_ESM.pdf]

A

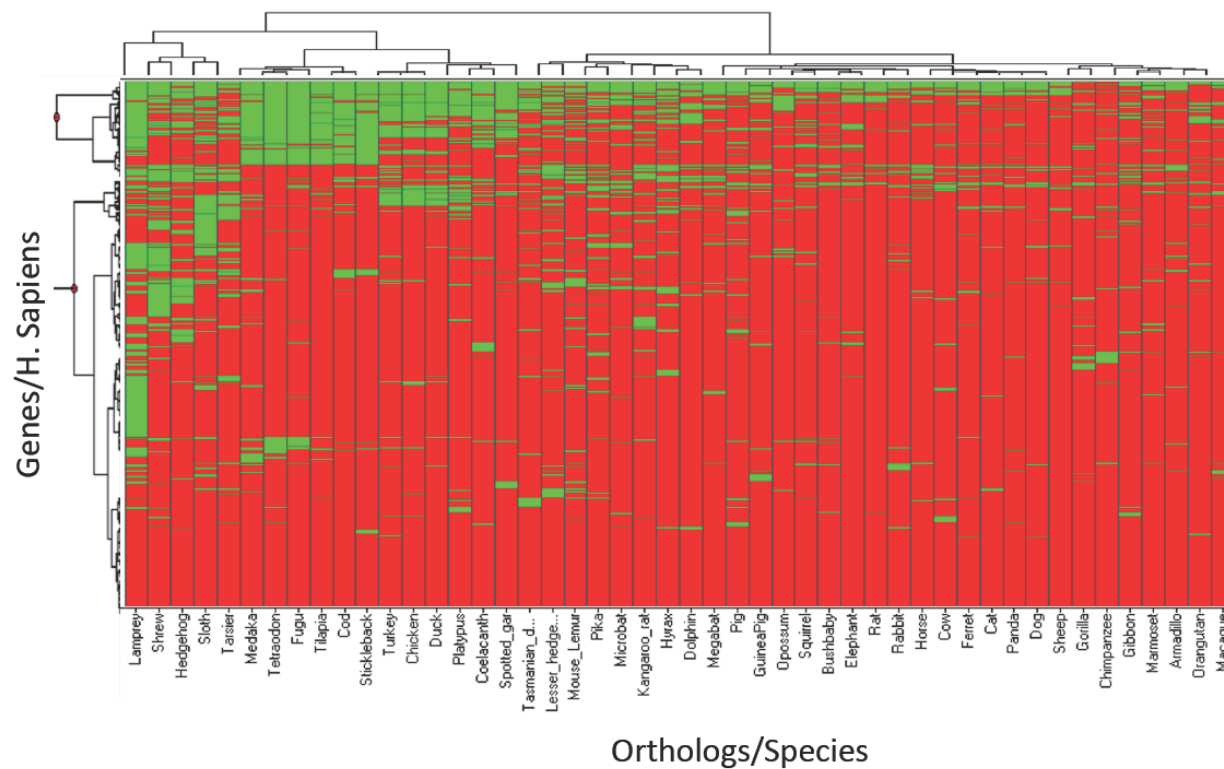

B

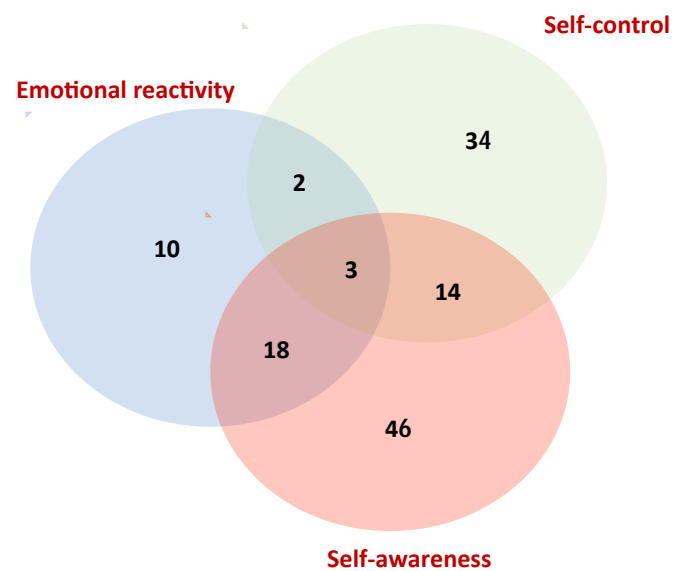

C

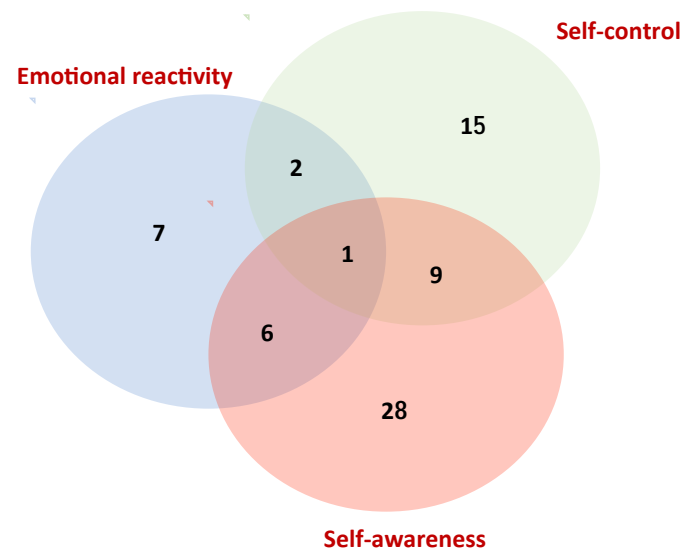

Fig. S4

Supplement: Supplementary file 5 — Supplementary Figure S4 [file 41380_2021_1097_MOESM5_ESM.pdf]

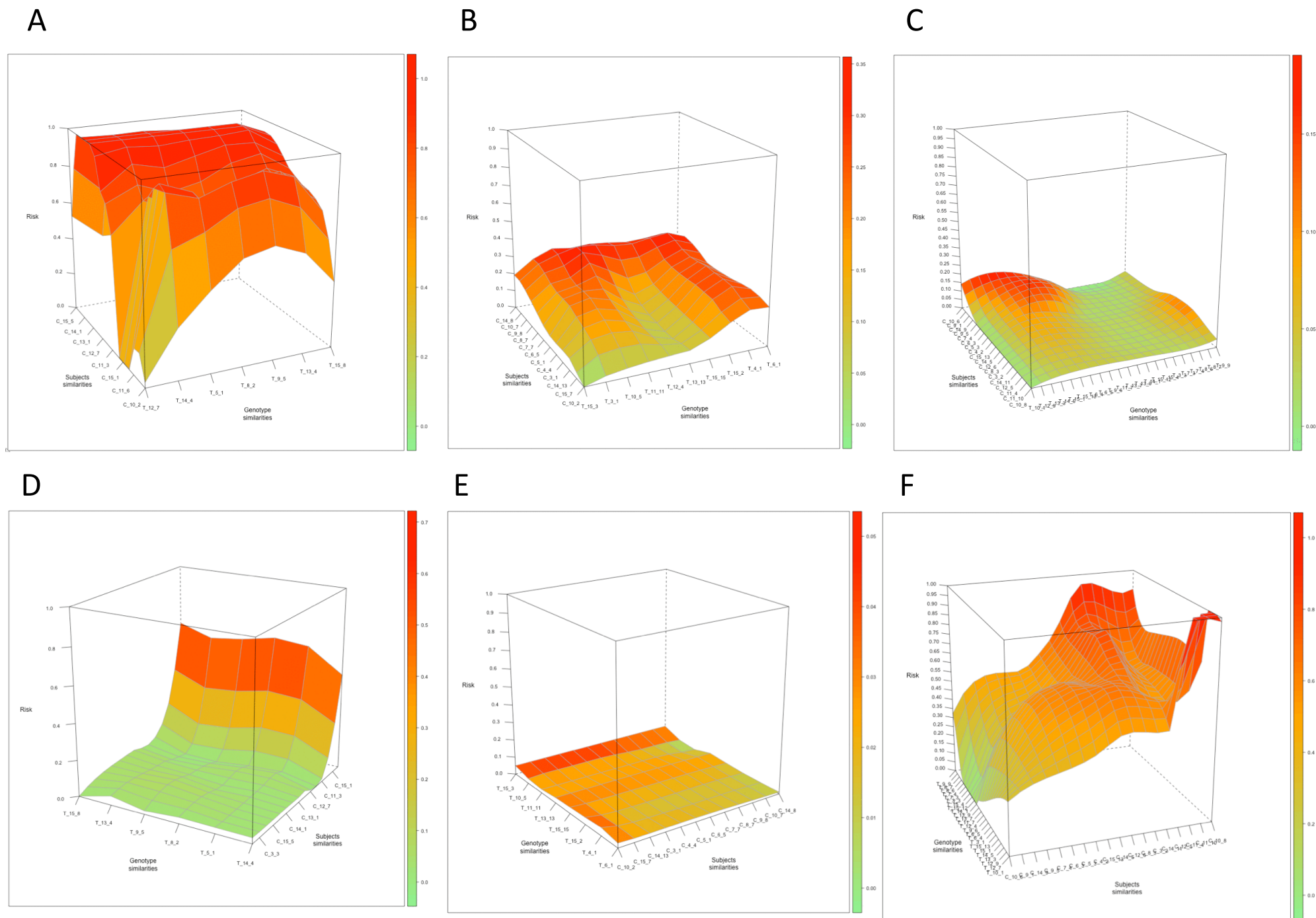

Fig. S5

Supplement: Supplementary file 6 — Supplementary Figure S5 [file 41380_2021_1097_MOESM6_ESM.pdf]

A

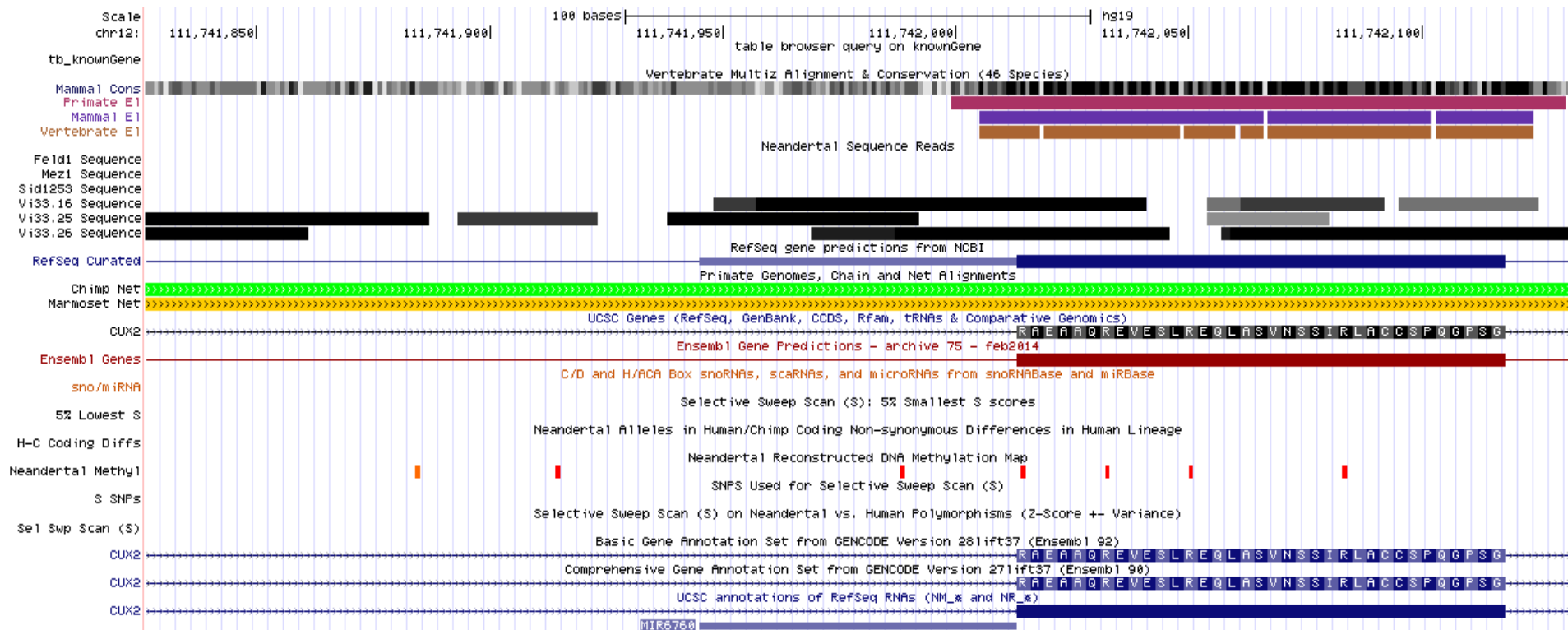

# B

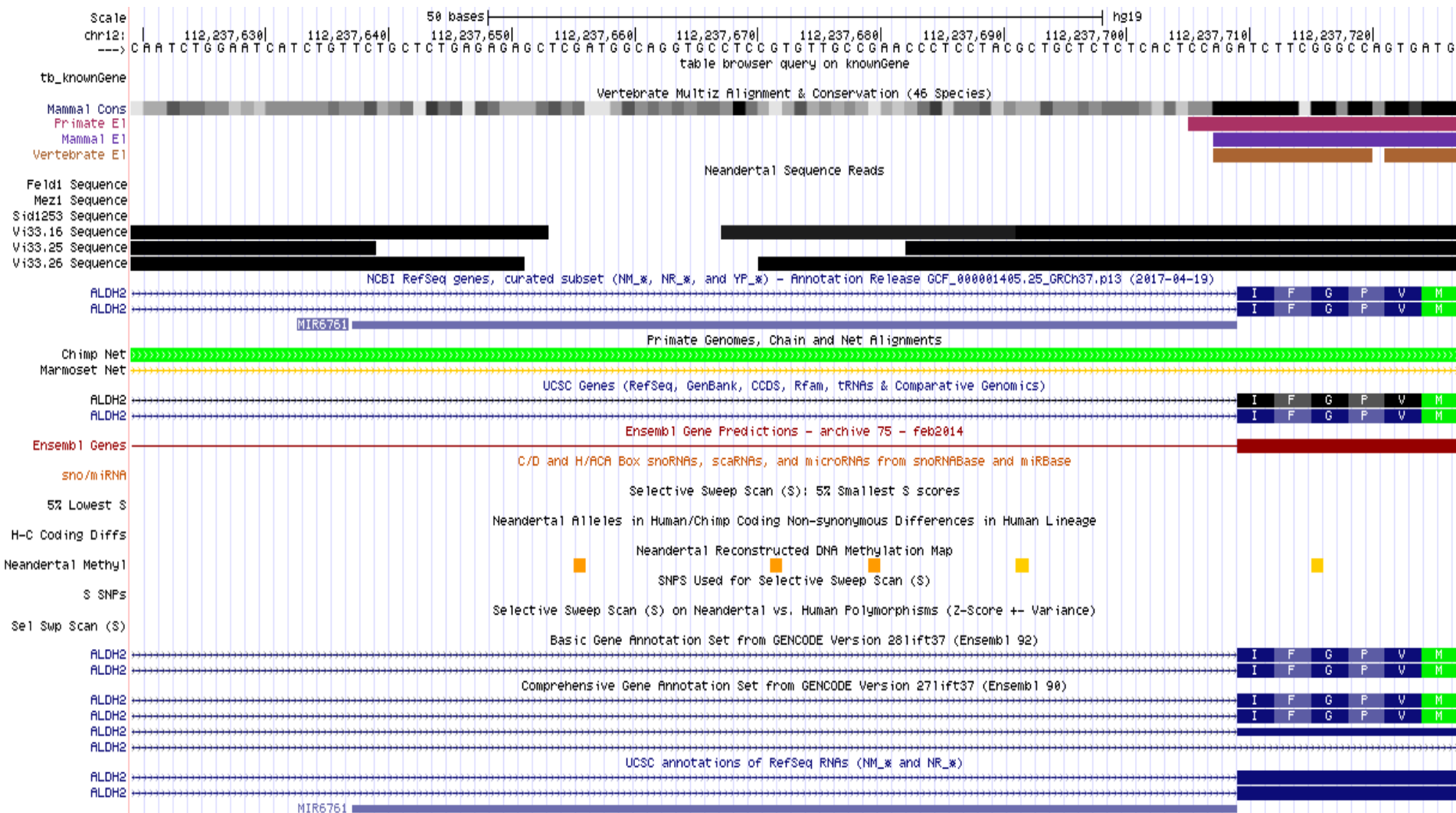

C

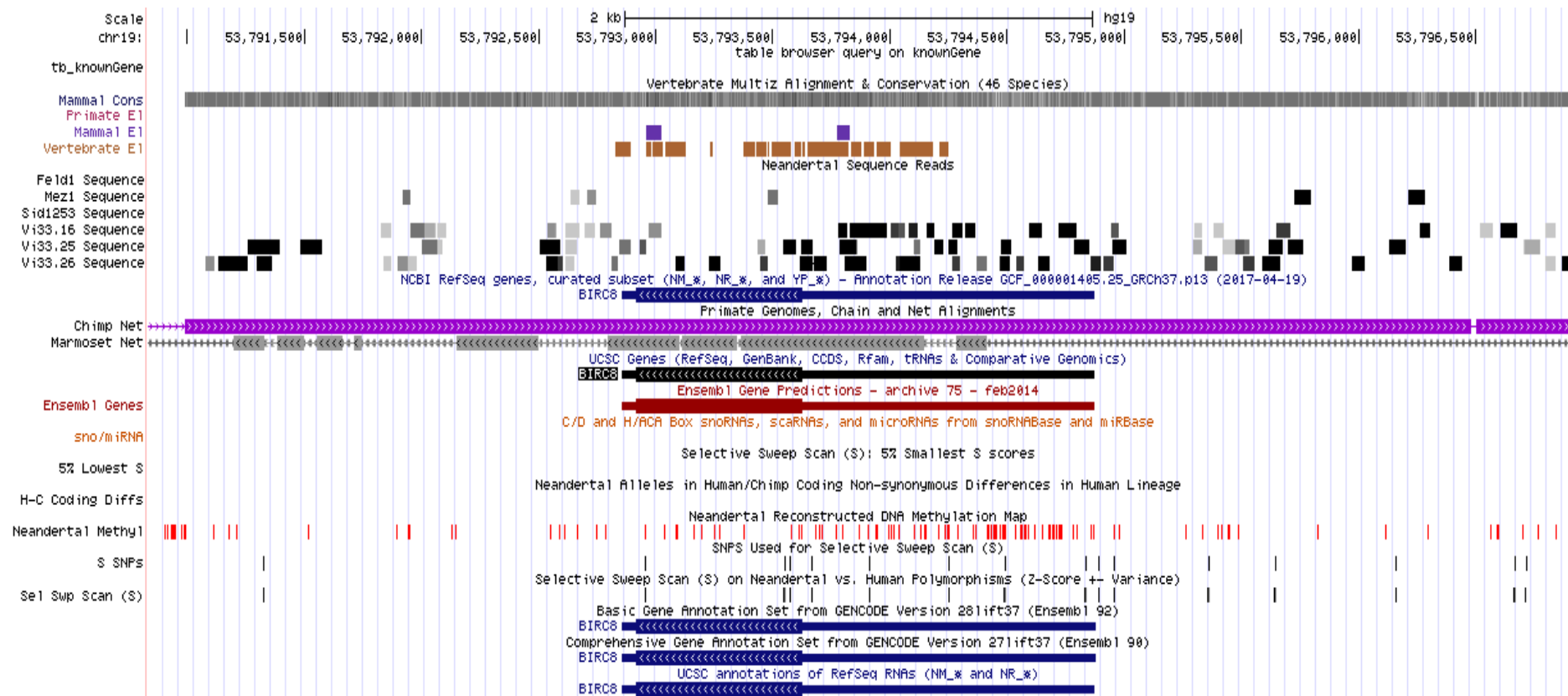

Fig. S6

Supplement: Supplementary file 7 — Supplementary Figure S6 [file 41380_2021_1097_MOESM7_ESM.pdf]
