# Supplementary material for: Evolution of genetic networks for human creativity
Source: Mol Psychiatry. 2021 Apr 21;27(1):354–76. doi: 10.1038/s41380-021-01097-y (PMC8960414; doi:10.1038/s41380-021-01097-y)
Supplement: Supplementary file 13 — Supplementary Table S6 [file 41380_2021_1097_MOESM13_ESM.docx]

|  | Count of genes | | | |
| --- | --- | --- | --- | --- |
| **Promoter DAF** | **Exon DAF** | | |  |
|  | *DAF >0,1* | *DAF<= 0,1* | *no results* | **Total** |
| *DAF >0,1* | 25 | 15 | 1 | **41** |
| *DAF<= 0,1* | 9 | 3 | 3 | **15** |
| *no results* | 2 | 2 | 8 | **12** |
| **Total** | **36** | **20** | **12** | **68** |

Table S6. Counts of lincRNA genes unique to modern Eurasian humans in terms of the DAF scores of their promoters and exons
