# Supplementary material for: Evolution of genetic networks for human creativity
Source: Mol Psychiatry. 2021 Apr 21;27(1):354–76. doi: 10.1038/s41380-021-01097-y (PMC8960414; doi:10.1038/s41380-021-01097-y)
Supplement: Supplementary file 14 — Supplementary Table S7 [file 41380_2021_1097_MOESM14_ESM.docx]

Table S7. Counts of lincRNA genes present in Neanderthals in terms of the DAF scores of their promoters and exons

|  | Count of genes | | | |
| --- | --- | --- | --- | --- |
| **Promoter DAF** | **Exon DAF** | | |  |
|  | *DAF >0,1* | *DAF<= 0,1* | *no results* | **Total** |
| *DAF >0,1* | 21 | 11 | 5 | **37** |
| *DAF<= 0,1* | 6 | 4 | 0 | **10** |
| *no results* | 5 | 1 | 6 | **12** |
| **Total** | **32** | **16** | **11** | **59** |
